# Genetic variants in primary cilia‐related genes associated with the prognosis of first‐line chemotherapy in colorectal cancer

**DOI:** 10.1002/cam4.6996

**Published:** 2024-02-09

**Authors:** Lei Qiu, Silu Chen, Shuai Ben, Jinxin Cui, Shan Lu, Rong Qu, Jinghuan Lv, Wei Shao, Qiang Yu

**Affiliations:** ^1^ Department of Gastroenterology The Affiliated Suzhou Hospital of Nanjing Medical University, Suzhou Municipal Hospital, Gusu School, Nanjing Medical University Nanjing Jiangsu China; ^2^ Department of Environmental Genomics, Jiangsu Key Laboratory of Cancer Biomarkers, Prevention and Treatment, Collaborative Innovation Center for Cancer Personalized Medicine Nanjing Medical University Nanjing China; ^3^ Department of Genetic Toxicology, The Key Laboratory of Modern Toxicology of Ministry of Education, School of Public Health Nanjing Medical University Nanjing China; ^4^ Department of Ophthalmology, Shanghai General Hospital, School of Medicine Shanghai Jiao Tong University Shanghai China; ^5^ Department of Pathology The Affiliated Suzhou Hospital of Nanjing Medical University, Suzhou Municipal Hospital, Gusu School, Nanjing Medical University Suzhou China

**Keywords:** colorectal cancer, genetic variants, *ODF2L*, primary cilia, prognosis

## Abstract

**Background:**

Primary cilia are antenna‐like organelles that conduct physical and chemical signals, which affect cell proliferation, migration, and differentiation. Some researchers have reported the correlation between primary cilia‐related genes and prognosis of colorectal cancer (CRC).

**Methods:**

The association between single nucleotide polymorphisms (SNPs) of primary cilia‐related genes and outcome after the first‐line chemotherapy was explored by the Cox regression model. Expression qualitative trait locus (eQTL) analysis was performed to explore the impact of SNPs on gene expression. Tumor Immune Estimation Resource and TISIDB databases were used for investigating the relevance between *ODF2L* and tumor infiltration immune cells and immunomodulators.

**Results:**

We identified that rs4288473 C allele of *ODF2L* had poor progression‐free survival (PFS) and overall survival (OS) of CRC patients in the additive model (adjusted HR_PFS_ = 1.39, 95% CI = 1.14–1.70, *p* = 1.36 × 10^−3^, and adjusted HR_OS_ = 1.31, 95% CI = 1.03–1.65, *p* = 2.62 × 10^−2^). The stratified analysis indicated that rs4288573 CC/CT genotype was involved with poor prognosis in the irinotecan‐treated subgroup (*P*_PFS_ = 1.03 × 10^−2^, *P*_OS_ = 3.29 × 10^−3^). Besides, *ODF2L* mRNA expression level was notably up‐regrated in CRC tissues. The C allele of rs4288573 was notably related to higher *ODF2L* mRNA expression levels based on eQTL analysis. Functionally, knockdown of *ODF2L* inhibited cell proliferation and decrease the chemoresistance of HCT‐116 and DLD‐1 cells to irinotecan.

**Conclusion:**

Our study indicates that rs4288573 in *ODF2L* is a potential predictor of the chemotherapy prognosis of CRC.

## 1 INTRODUCTION

Colorectal cancer (CRC) represents approximately 10% of all cancers and is the second most common cause of cancer death.^1^ Besides, the deaths and incidence are increasing among diagnosed at young people.^2^, ^3^ The main treatment for CRC is chemotherapy, radiotherapy, targeted therapy, and immunotherapy. However, chemotherapy drug resistance was the major reason for therapy defeat in CRC.^4^ Oxaliplatin and irinotecan are both cytotoxic drugs, which are peripheral neuropathy, and gastrointestinal toxicity, respectively.^5^ FOLFIRI (fluorouracil, leucovorin combined with irinotecan) and FOLFOX (fluorouracil, leucovorin combined with oxaliplatin) therapy reduced adverse drug reactions and significantly prolonged metastatic CRC patient survival.^5^, ^6^ Unfortunately, the clinical efficacy of colorectal cancer chemotherapy is restricted to drug resistance.^7^

Clinical trials showed that molecular subtypes of CRC affect patient survival.^8^ According to the molecular characteristics of the tumor, the corresponding chemotherapy regimen can improve the overall survival (OS) rate.^9^ Prognostic markers are related to the prognosis of chemotherapy regimens. *KRAS* mutation predicts non‐response to EGFR‐targeted drugs is a breakthrough in CRC molecular markers.^10^ Single nucleotide polymorphisms (SNPs) in the folic acid metabolic pathway and DNA repair system genes were related to prediction of CRC chemotherapy efficacy.^11^, ^12^

Primary cilia are tentacle‐like structures on the surface of the cells, regulating the signal conduction between cells and the matrix, which affects cell proliferation, migration, and differentiation.^13^ Additionally, primary cilia were associated with several important paracellular pathways between tumor microenvironment (TME) cells.^14^, ^15^ Signaling among cancer cells and cells in TME influences tumor chemotherapy response.^15^ The absence of primary cilia promotes thyroid cancer development.^16^ Besides, primary cilia are essential for controlling tumor invasion and the reduction of primary cilia also leads to the excessive proliferation of bile duct cells.^17^, ^18^ Studies also revealed that knockdown *TTLL3* and *TTLL8*, primary cilia‐related genes, lessened the count of primary cilia, which leads to accelerated colorectal epithelial cell proliferation and CRC in mice models.^19^ A recent study proposed that the absence of primary cilia enhances BRAF/MAPK pathway, thereby affecting the resistance to therapy of CRC.^20^

Centrioles coordinate the primary microtubule organizing center of the cell and template the formation of cilia.^21^ Some studies have suggested that human microsatellite instable and BRAF‐V600E‐mutation CRC with a lethal rhabdomyolic phenotype are characterized by centrosome functional inactivation and a correlation between CROCC gene variant and centrosome abnormality has been proposed.^22^ Besides, the same team discovered that the relationship between histopathologic type treatment and prognosis of colorectal cancer.^23^ Some studies have shown that mismatch repair genes (MMR) polymorphisms may affect treatment outcomes of CRC patients.^24^

Currently, some researchers have explored the predictive value of genetic variants in primary cilia‐related genes on colorectal cancer. In our research, we further explored the function of primary cilia‐related genes. We also investigated the effect of genetic variants in CRC prognosis.

## 2 MATERIALS AND METHODS

### 2.1 Study population

Our research recruited 344 CRC patients from the Affiliated Nanjing First Hospital and the First Affiliated Hospital of Nanjing Medical University in September 2010. Due to incomplete information on 19 patients, we only performed the analysis on the remaining 325 patients. The clinical information of 325 patients has been demonstrated in our previous study.^12^, ^25^ Among them, 137 patients received irinotecan‐based chemotherapy and 188 patients received oxaliplatin‐based chemotherapy. Besides, our cohort excluded patients receiving radiation therapy. Progress‐free survival (PFS) was the moment from the start of chemotherapy to progressive disease or death. The definition of OS was counted from the first day of chemotherapy to death or miss record. Complete response (CR), partial response (RR), stable disease (SD) and progressive disease (PD) together make up the tumor response. The disease control rate (DCR) was defined as CR, PR and SD proportion. The study was approved by the Institutional Review Board of Nanjing Medical University and the written informed consent was provided by all participants.

### 2.2 The selection of primary cilia‐related genes and SNPs

First, we found four genes that inhibit primary cilia structure, fifteen genes that promote primary cilia structure formation, and eight primary cilia‐related genes on PubMed. Second, SNPs located in ±2 kb flanking regions of these genes were screened by the Chinese Han population in Beijing (CHB) population data from the 1000 Genomes Project (March 2012). The following criteria were used to screen SNPs: (a) minor allelic frequency (MAF) ≥ 0.05, and (b) Hardy–Weinberg equilibrium (HWE) exact *p‐*value ≥0.01; Third, we kept tagging SNPs (*r* ^2^ ≤ 0.8) in low linkage disequilibrium (LD) with HaploView 4.2 software. We took the genomic DNA from CRC patients and used the Qiagen Blood kit (Qiagen). Specific SNP genotyping methods refer to our previous article.^26^

### 2.3 Functional analysis

Then function analysis of SNPs was performed by 3D SNP, HaploReg v4.1 and RegulomDB. The Retrieval of Interacting Genes constructed a protein–protein interaction network (PPI). We used online tools including RegulomDB, 3D SNP and HaploReg v4.1 to distinguish potentially representative functional SNPs. Besides, the secondary structure of the candidate SNP was predicted by the RNA fold website. TISIDB database is a web platform to explore the association between tumors and the immune system. Detail information for function analysis are available in Data S1.

### 2.4 Expression analysis

The Cancer Genome Atlas (TCGA), Gene Expression Omnibus (GEO), and in‐house databases compared the mRNA expression levels (log2 transformed) between cancer tissues and adjacent normal tissues. The Genotype‐Tissue Expression (GTEx) project dataset was accustomed to performing the expression qualitative trait locus (eQTL) analysis and evaluating the correlation between genotypes of SNP and gene expression. To calculate the association between genotypes of SNPs and alternative splicing (AS) in CRC, we also performed splicing quantitative trait loci (sQTL) analysis by CancerSplicingQTL database. AS events were evaluated by a percent‐splice‐in (PSI) value, which is the ratio of normalized read counts indicating the inclusion of a transcript element over the total normalized reads for that event.^27^ The Tumor Immune Estimation Resource (TIMER) database explored the association between *ODF2L* expression and immune infiltration.

### 2.5 Statistical analysis

Cox regression models were estimated the role of genetic variants on PFS and OS in CRC patients undergoing chemotherapy. Logistic regression model was performed to explore the influence of the candidate SNPs on DCR. To reduce the probability of false positives, *p*‐value adjustment was corrected by false discovery rate (FDR). Student's *t‐*test was utilized to compare the differential expressions of mRNA from the TCGA and GEO databases. Survival curves were calculated by the Kaplan–Meier method. The association between candidate SNPs and survival of CRC was further validated in the UK biobank and the TCGA cohorts. R software (version 3.6.3) and PLINK 1.09 were used for primary statistical analysis. *p‐*value <0.05 was considered statistically significant.

## 3 RESULTS

### 3.1 Primary cilia‐related genes and candidate SNPs selection

**FIGURE 1:**
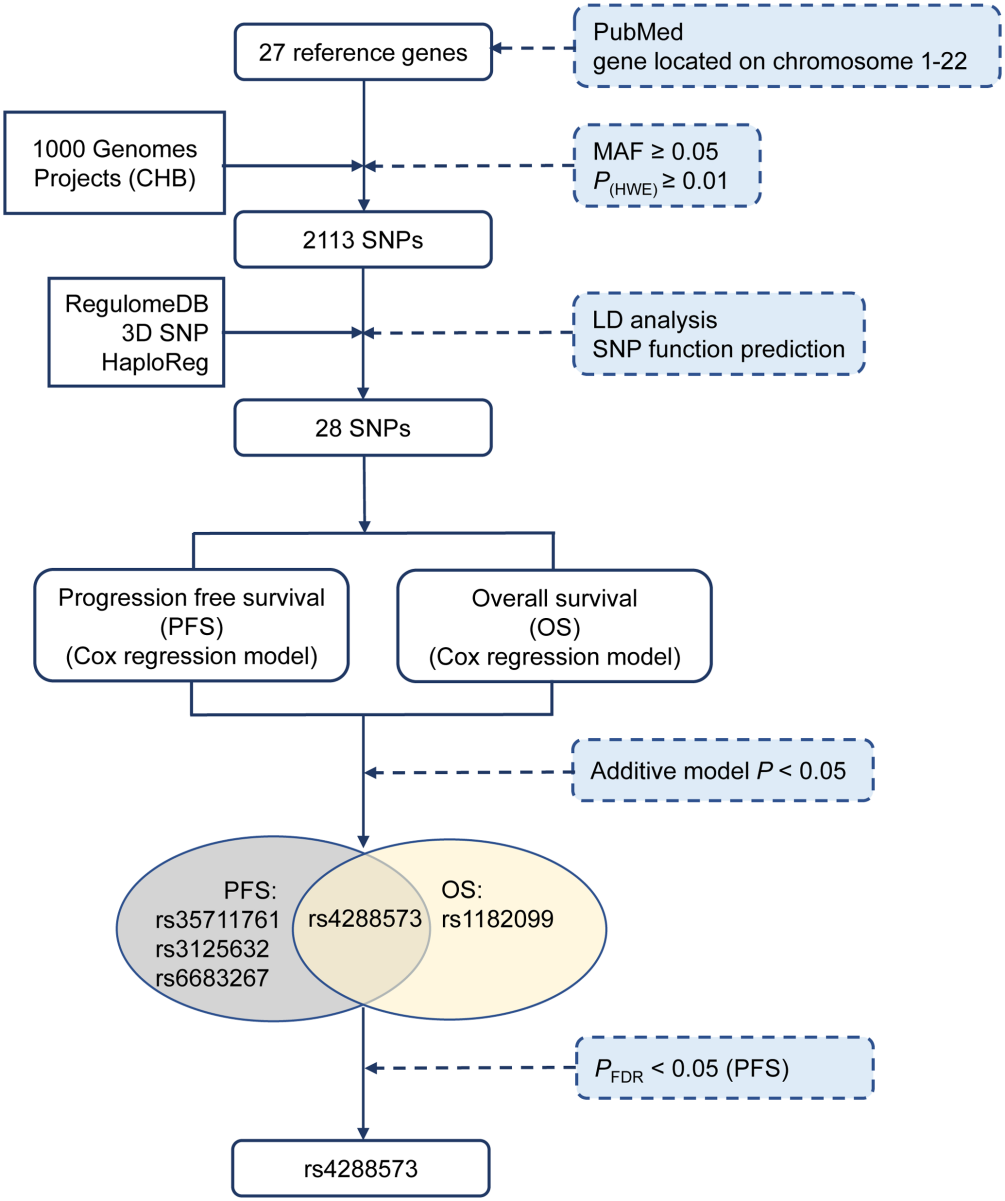
The Flow diagram of selected SNPs in primary‐related genes. CHB, Han Chinese in Beijing; MAF, minor allele frequency, HWE, Hardy–Weinberg Equilibrium; LD, linkage disequilibrium; SNP, single‐nucleotide polymorphism; PFS, progression‐free survival; OS, overall survival; FDR, false discovery rate.

As shown in Figure 1, a total of 27 primary cilia‐related genes that were reported by previous studies were selected in this study (Table S1). These genes presented significantly differential expression levels between CRC and adjacent normal tissues in the TCGA database (Figure **S1**A). Figure **S1**B shows the PPI network of the candidate genes. 2113 SNPs in these 27 genes remained for further LD analysis and functional annotation. Ultimately, 28 SNPs were selected to investigate their relationships with CRC survival after chemotherapy.

### 3.2 Association of rs4288573 in *ODF2L* and colorectal cancer survival

The relationship between 28 SNPs and PFS and OS of CRC was analyzed in the additive model after adjusting for sex, age, smoking status, and drinking status (Table S2). Only four SNPs were significantly associated with PFS and two SNPs were remarkably associated with OS of CRC patients after first‐line chemotherapy. After FDR correction, rs4288573 in *ODF2L* was markedly related to decreased PFS in CRC (*P*_FDR_ = 3.82 × 10^−2^). Therefore, we focused on rs4288573 in *ODF2L* for further analysis.

**FIGURE 2:**
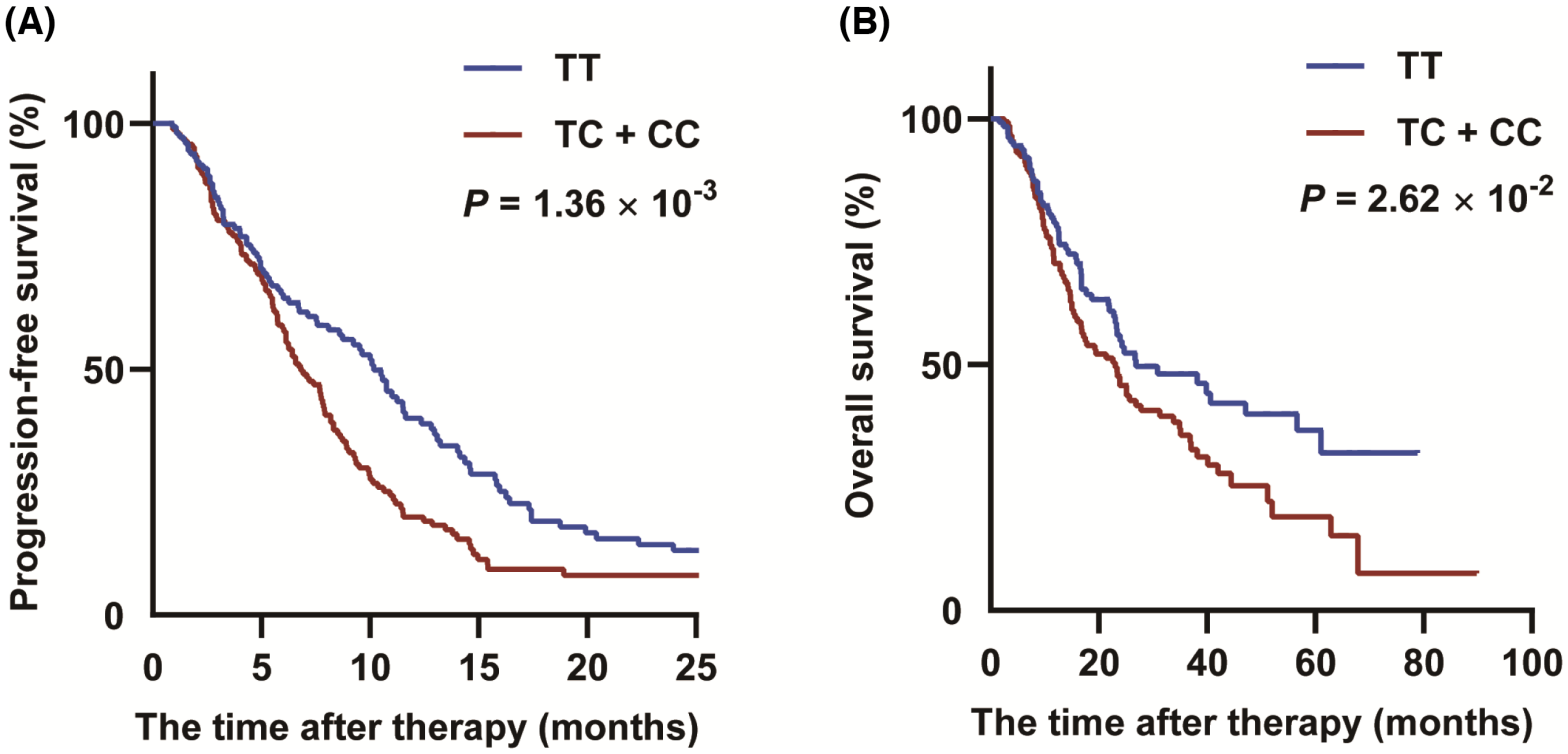
Kaplan–Meier curve of rs4288573 in the dominant model. (A) Kaplan–Meier curves of progression‐free survival (PFS) by Cox regression model in colorectal cancer (CRC) patients. (B) Kaplan–Meier curves of overall survival (OS) by Cox regression model in CRC patients.

Besides, we explored the association between rs4288573 and survival in different genetic models (Table 1). SNP rs4288573 in *ODF2L* was related to poor PFS of CRC patients (adjusted HR = 1.39, 95% CI = 1.14–1.70, *p* = 1.36 × 10^−3^ for the additive model; adjusted HR = 1.42, 95% CI = 1.09–1.86, *p* = 1.05 × 10^−2^ for the dominant model; HR = 1.78, 95% CI = 1.18–2.68, *p* = 6.02 × 10^−3^ for the recessive model). Similar results were investigated in OS (adjusted HR = 1.31, 95% CI = 1.03–1.65, *p* = 2.62 × 10^−2^ for the additive model; HR = 1.46, 95% CI = 1.05–2.03, *p* = 2.59 × 10^−2^ for the dominant model). Kaplan–Meier curves indicated that CC/CT genotype was meaningfully related to poor prognosis compared with the TT genotype (Figure 2).

**TABLE 1:** Association between rs4288573 and survival of colorectal cancer patients in four genetic models.

Model	*N* (%)	PFS		OS	
		HR (95% CI)^a^	*p*‐value^a^	HR (95% CI)^a^	*p*‐value^a^
TT	150 (46.44%)	1.00		1.00	
TC	135 (41.80%)	1.32 (0.99–1.75)	5.80 × 10^−2^	1.42 (1.00–2.02)	5.01 × 10^−2^
CC	38 (11.76%)	2.04 (1.32–3.15)	1.42 × 10^−3^	1.60 (0.95–2.70)	7.93 × 10^−2^
Additive model		1.39 (1.14–1.70)	1.36 × 10^−3^	1.31 (1.03–1.65)	2.62 × 10^−2^
Dominant model		1.42 (1.09–1.86)	1.05 × 10^−2^	1.46 (1.05–2.03)	2.59 × 10^−2^
Recessive model		1.78 (1.18–2.68)	6.02 × 10^−3^	1.36 (0.83–2.22)	2.23 × 10^−1^

Abbreviations: CI, confidence interval; HR, hazard ratio; PFS, progression‐free survival; OS, overall survival.

^a^ Adjusted for sex, age, smoking status, and drinking status in the Cox regression model.

Moreover, we further explored the association between rs4288573 and survival in different genetic models in the TCGA database excluding Asian population for validation (Table S3). We also investigated the association between rs4288573 and OS and CRC‐specific survival (CSS) in the UK Biobank (UKBB) cohort from an online website SUMMER (Figure S2). Based on the above validation cohorts, we did not observe a statistically significant between rs4288573 and survival time of CRC patients. These findings suggest that considerable heterogeneity exists in the effect of rs4288573 between different ethnic groups.^28^

### 3.3 Stratification and interaction analyses of rs4288573 in colorectal cancer survival

Stratified analysis was performed to analyze the association between rs4288573 and clinical characteristics after first‐line chemotherapy in the dominant model (Table 2). We found rs4288573 CC/CT genotypes had a dramatic influence on reducing PFS and OS of CRC patients of male, smokers, drinkers, patients of Dukes stage D, metastatic ≤2 and patients with irinotecan‐based chemotherapy.

**TABLE 2:** Stratified analysis of the association between rs4288573 and survival of colorectal cancer in the dominant model.

Variables	PFS		OS	
	HR (95% CI)^a^	*p*‐value^a^	HR (95% CI)^a^	*p*‐value^a^
Age				
≤60	1.52 (1.05–2.21)	2.73 × 10^−2^	1.53 (0.96–2.44)	7.44 × 10^−2^
>60	1.35 (0.90–2.04)	1.46 × 10^−1^	1.20 (0.74–1.95)	4.62 × 10^−1^
Sex				
Male	1.45 (1.04–2.02)	2.90 × 10^−2^	2.02 (1.32–3.09)	1.23 × 10^−3^
Female	1.36 (0.85–2.17)	1.93 × 10^−1^	0.83 (0.47–1.49)	5.41 × 10^−1^
Smoking status				
Yes	1.84 (1.15–2.94)	1.11 × 10^−2^	2.50 (1.36–4.58)	3.09 × 10^−3^
No	1.21 (0.86–1.70)	2.70 × 10^−1^	1.08 (0.72–1.64)	7.03 × 10^−1^
Drinking status				
Yes	1.72 (1.03–2.89)	4.00 × 10^−2^	2.09 (1.04–4.18)	3.75 × 10^−2^
No	1.31 (0.94–1.80)	1.06 × 10^−1^	1.27 (0.86–1.87)	2.31 × 10^−1^
Tumor site				
Rectum	1.76 (1.14–2.73)	1.10 × 10^−2^	1.32 (0.80–2.17)	2.83 × 10^−1^
Colon	1.42 (0.99–2.03)	5.63 × 10^−2^	1.58 (1.00–2.49)	4.78 × 10^−2^
Tumor grade				
Moderate and well	1.31 (0.97–1.78))	8.27 × 10^−2^	1.48 (1.00–2.18)	4.78 × 10^−2^
Poor	2.38 (1.26–4.48)	7.27 × 10^−3^	1.62 (0.80–3.29)	1.78 × 10^−1^
Dukes stage				
C	1.89 (0.42–8.52)	4.07 × 10^−1^	1.11 (0.06–19.89)	9.42 × 10^−1^
D	1.42 (1.07–1.87)	1.38 × 10^−2^	1.47 (1.05–2.06)	2.59 × 10^−2^
Metastasis				
≤2	1.47 (1.08–2.00)	1.49 × 10^−2^	1.64 (1.01–2.40)	1.33 × 10^−2^
>2	1.21 (0.59–2.49)	6.05 × 10^−1^	0.82 (0.37–1.80)	6.17 × 10^−1^
Treatment				
Oxaliplatin	1.27 (0.87–1.85)	2.18 × 10^−1^	1.11 (0.71–1.74)	6.34 × 10^−1^
Irinotecan	1.68 (1.13–2.51)	1.03 × 10^−2^	2.14 (1.29–3.55)	3.29 × 10^−3^

Abbreviations: CI, confidence interval; HR: hazard ratio; PFS, progression‐free survival; OS, overall survival.

^a^ Adjusted for sex, age, smoking status, and drinking status in the Cox regression model.

Moreover, interaction analysis supported a notable interaction effect between rs4288573 and smoking status (*P*_interaction_ = 4.43 × 10^−2^) on CRC patients (Figure S3). Compared with individuals in non‐smokers with the TT genotype, the CC/CT genotypes present a 1.72‐fold elevated risk of poor survival in smokers.

### 3.4 Association between rs4288573 and colorectal cancer prognosis undergoing irinotecan‐based chemotherapy

**FIGURE 3:**
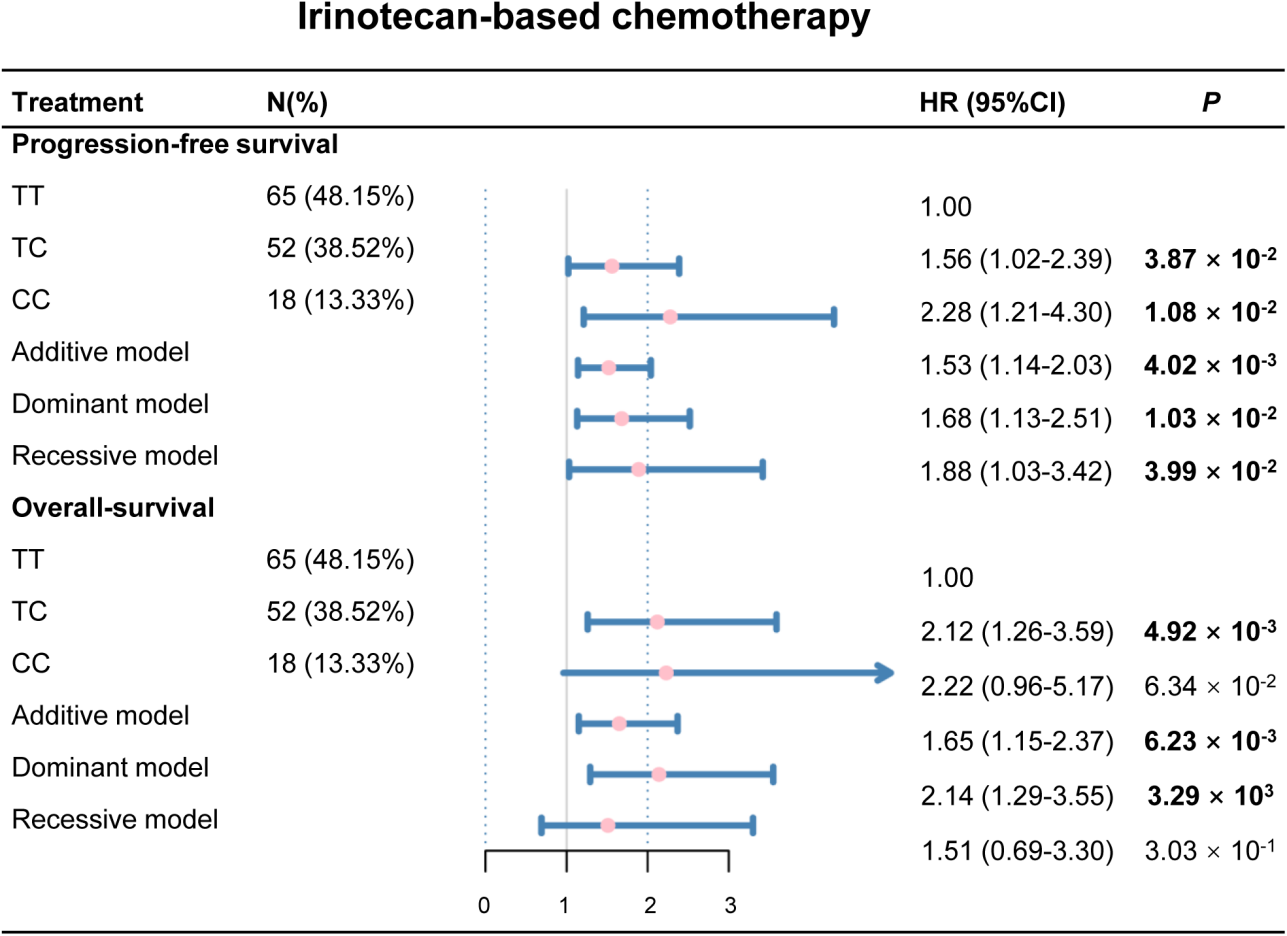
The association between rs4288573 and prognosis of patients with irinotecan‐based chemotherapy in four genetic models.

Based on the above stratified analysis, we found that rs4288573 had different effects on the prognosis of CRC patients with two chemotherapies. Therefore, we explored the association between rs4288573 and the prognosis of CRC with different chemotherapy through four genetic models. We found that rs4288573 was weakly associated with the efficacy of the oxaliplatin‐based chemotherapy (Figure S4), while it was significantly associated with the irinotecan‐based chemotherapy (Figure 3). Among these patients receiving irinotecan‐based chemotherapy, the four genetic of rs4288573 were associated with reduced PFS after adjustment (*p* < 0.05 for four genetic models), and rs4288573 was also strongly related to poor OS after adjustment in CRC patients in the additive model and the dominant model (*p* < 0.05, respectively).

Our results showed a poor prognosis in patients with rs4288573 CC/CT genotypes and treated with irinotecan‐based chemotherapy. Therefore, we further analyzed the relevance between rs4288573 and clinical characteristics in patients with irinotecan‐based chemotherapy in the dominant model. After adjustment, a poor prognosis was observed among patients with CC/CT genotypes in males, smokers, rectal cancer, moderate and well tumor grade, Dukes stage D and metastasis ≤2 (*p* < 0.05, Table S4). Furthermore, SNP rs4288573 CC/CT genotypes were considerably significant between poor DCR among males and smokers (adjusted HR = 3.77, 95% CI = 1.34–11.88, *p* = 1.58 × 10^−2^, adjusted HR = 5.77, 95% CI = 1.48–29.75, *p* = 1.87 × 10^−2^, respectively; Figure S5).

### 3.5 In silico function prediction of rs4288573 in *ODF2L*

**FIGURE 4:**
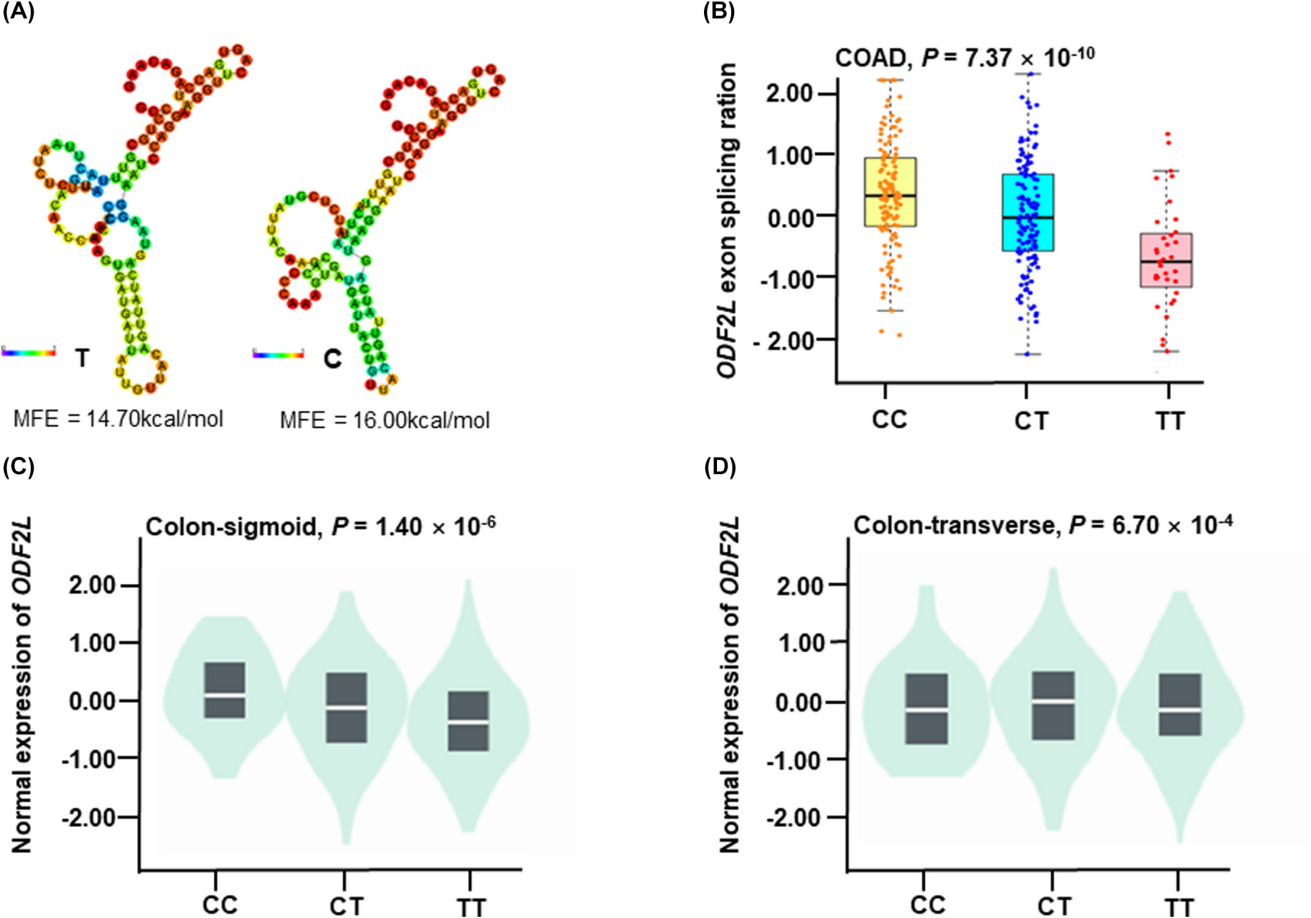
Functional annotation for rs4288573. (A) Secondary structure of the rs4288573. (B) The correlation between rs4288573 and the splicing events of *ODF2L* in colon cancer is based on the CancerSplicingQTL database. (C, D) The eQTL analysis between rs4288573 CC/CT and *ODF2L* mRNA expression in colon‐transverse and colon‐sigmoid tissues from the GTEx database.

We constructed a comprehensive functional prediction of rs4288573 utilizing online bioinformatic tools, including RegulomDB, HaploReg, and 3D SNP (Table S5). SNP rs4288573 was predicted to have potential functions of Promote histone marks, Enhancer histone marks, DNAse, Proteins bound, Motifs changed and Selected eQTL hits. The 3D SNP score and RegulomDB score were 122.98 and 1f, respectively. We also predicted the RNA secondary structure and the change in the minimum free energy (MFE) of the T allele to the C allele, the MFE decreased from −14.70 kcal/mol to −16.00 kcal/mol (Figure 4A).

### 3.6 The sQTL analysis and eQTL analysis

The CancerSplingQTL database was used to evaluate the relationship between the genotypes of rs4288573 and AS events of *ODF2L* in CRC. CC and CT genotypes exhibited a higher splicing ratio than the TT genotype in *ODF2L* (*p* = 7.37 × 10^−10^, Figure 4B). To explore the biological relevance of rs4288573 and its corresponding mRNA expression, we next investigate its eQTL effect by the GTEx database. Genotypes of rs4288573 presented a significant association with *ODF2L* mRNA expression in both colon‐sigmoid samples (*p* = 1.40 × 10^−6^, Figure 4C) and colon‐transverse samples (*p* = 6.70 × 10^−4^, Figure 4D).

### 3.7 Aberrant *ODF2L* expression in colorectal cancer

**FIGURE 5:**
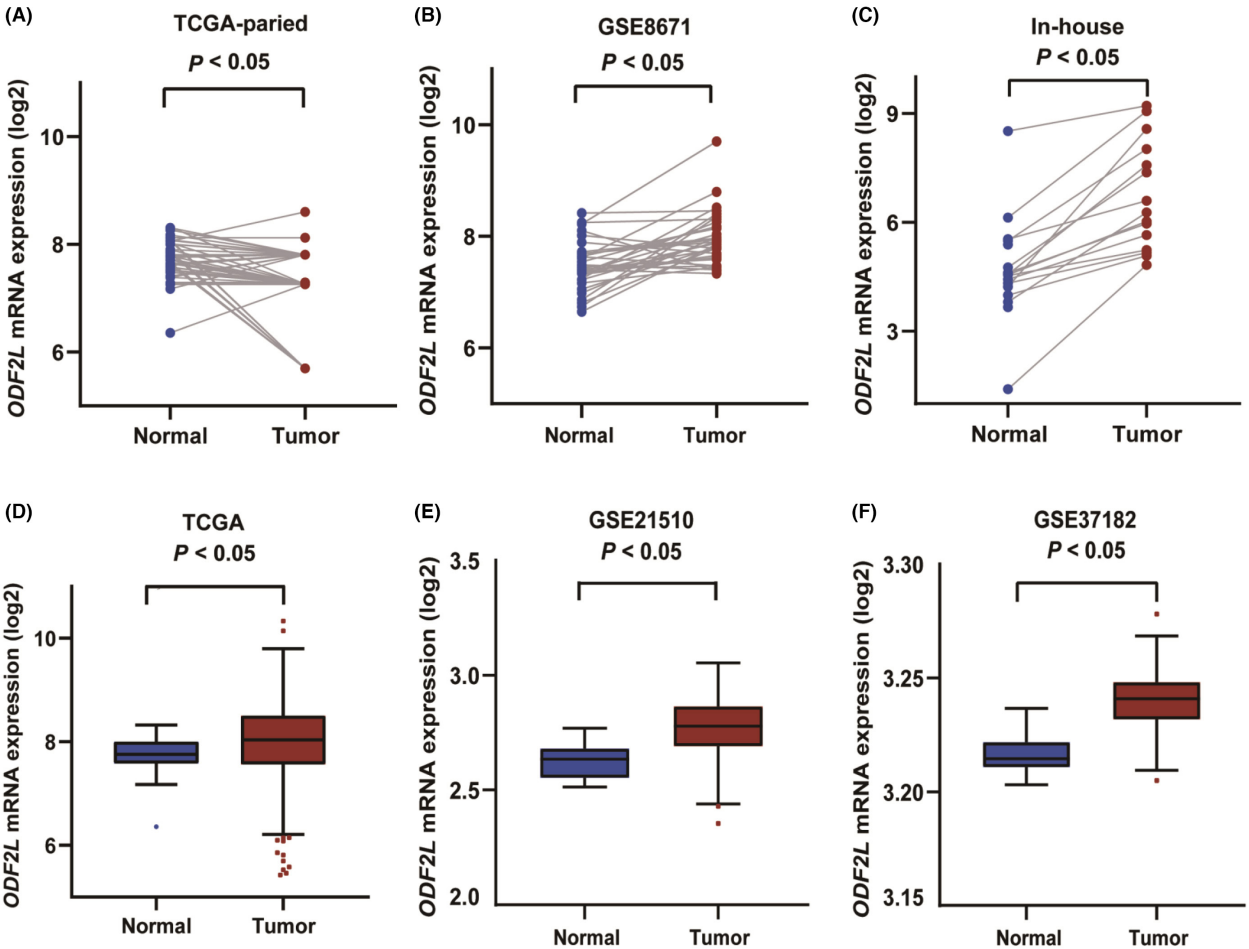
*ODF2L* is significantly overexpressed in colorectal cancer tissues than adjacent normal tissues. (A, D) *ODF2L* mRNA expression levels in the TCGA database; (B, E, F) in the GEO database (GSE8671, GSE21510, GSE37182); (C) in‐house database.

*ODF2L* mRNA expression levels were explored between CRC and normal tissues in the TCGA, GEO, and in‐house databases. Compared to adjacent normal tissues, paired CRC tissues presented increased mRNA expression levels of *ODF2L* (*p* < 0.05, Figure 5A–C). Unpaired tissue samples were also analyzed in the same database. As illustrated in Figure 5D–F, CRC tissues exhibited markedly higher mRNA expression levels of *ODF2L* than adjacent normal tissues (*p* < 0.05). Additionally, the *ODF2L* expression level was significantly increased in the aged (≤60), in overweight (BMI > 28), and in colon cancer (*p* < 0.05, Figure S6).

### 3.8 Association of *ODF2L* expression and Immune infiltration

TIMER database revealed *ODF2L* expression was positively involved with infiltrating levels of B cell, CD8+ T cell, Macrophage, Neutrophil, and Dendritic Cell in colorectal adenocarcinoma (COAD), while a significant association was only found between the expression of *ODF2L* and CD8+ T Cell, Neutrophil, Dendritic Cell in rectal adenocarcinoma (READ) (*p* < 0.05, Figure S7A). Besides, there was a remarkable association between the copy numbers of *ODF2L* and immune cell infiltration levels in COAD (Figure S7B). Besides, *ODF2L* was associated with immune stimulators, including ENTPD1 in COAD and READ (*p* < 0.05, Figure S8A). *ODF2L* expression was also positively related to immune inhibitors, including BTLA, KDR, TGFBR1, and VTCN1 in COAD, and CD274, KDR, VTCN1, and TGFBR1 in READ (*p* < 0.05, Figure S8B). Meanwhile, *ODF2L* expression was positively related to chemokine, such as CCL20 and CXCL11 in COAD, CXCL5, CXCL6 CXCL8 and CXCL9, CXCL10 and CXCL13 in READ (*p* < 0.05, Figure S9).

### 3.9 Effect of *ODF2L* on colorectal cancer cell proliferation and chemotherapy resistance

Western blot and quantitative real‐time PCR (qRT‐PCR) were used to estimate the mRNA expression of ODF2L levels in six colon cancer cell lines. ODF2L expression was higher in HCT‐116 and DLD‐1 than other cells (Figure S10A). For that reason, HCT‐116 and DLD‐1 were selected for next research. We knockdown *ODF2L* in HCT‐116 and DLD‐1 cells by transient transfection of ODF2L siRNA (si‐*ODF2L*). Western blot verified the change of ODF2L expression level (Figure S10B). Then we examined the role of *ODF2L* on cell proliferation in two cell lines. Knockdown *ODF2L* remarkably reduced the rate of cell proliferation (Figure S11A,B). To explore the affection of *ODF2L* gene on cellular sensitivity to irinotecan, we transfected si‐*ODF2L* into HCT‐116 and DLD‐1 cells. Our findings showed that inhibition of *ODF2L* significantly decreases cell activity of HCT‐116 and DLD‐1 cells when compared with control cells (Figure S10C,D). These results indicated that knockdown of *ODF2L* was sufficient to decreased the chemoresistance of HCT‐116 and DLD‐1 cells to irinotecan.

## 4 DISCUSSION

We found a significant correlation between rs4288573 in *ODF2L*, the primary cilia‐related gene, and the poor prognosis of CRC. SNP rs4288573 T > C had a considerable influence on the reduced PFS and OS of CRC patients. The rs4288573 T > C increased the expression of mRNA of *ODF2L*, which itself inhibits the production of primary cilia.^29^ It has been previously declared that the deficiency of primary cilia promotes intestinal inflammation and cancer development and is a potential molecular marker for CRC.^30^ Patients with high cilia frequency have considerably longer OS compared to low cilia frequency in CRC.^31^

Currently, drug resistance in cancer is one of the major dilemmas faced by researchers. Studies have indicated that many drug targets are part of the signaling pathways regulated by primary cilia.^32^ Scientists have found that loss of cilia can cause drug resistance in cell lines.^33^ Primary cilia contain specific receptors and ion channel proteins that connect motor, mechanical, and chemical stimuli to intracellular signals and are related to various signaling pathways that regulate various processes of cell development and maintain tissue homeostasis.^34^, ^35^ More studies supported the correlation between primary cilia and cancer progression.^13^ The number of primary cilia is substantially decreased in breast cancer, pancreatic, and CRCs.^31^, ^36^, ^37^ The same conclusion was observed in prostate cancer, where elevated expression of genes that inhibit primary cilia formation, thereby promoting tumor cell proliferation and tumor growth.^38^ Loss of primary cilia was strongly associated with tumor development and tumor chemotherapy resistance.^39^ Our study also indicated that CRC patients receiving irinotecan‐based chemotherapy with *ODF2L* variants were less sensitive to chemotherapy and had worse prognosis.

Functional annotations revealed that rs4288573 had multiple biological functions. SNP rs4288573 T > C increased the expression of *ODF2L*. AS event is a gene expression regulation mechanism that allows a single gene to produce multiple mRNA products, thus increasing the complexity of proteomics. Therefore, AS events are involved in cell and tissue development.^40^ AS regulated highly diverse processes, such as tissue‐specific and species‐specific cell differentiation cancer, and so on.^41^ In our study, we assessed the correlation between SNP rs4288573 that influence AS events of *ODF2L*. Interestingly, we inferred that CC genotype of rs4288573 may increase the ODF2L exon splicing ration, thus increasing *ODF2L* expression and reducing the number of primary cilia, leading to a poor prognosis of CRC.

The prognosis of CRC may be closely related to diet and lifestyle.^42^ Therefore, stratified analysis was accustomed to investigating the correlation between specific lifestyles and the CRC prognosis. Studies have found that smoking has an adverse effect on CRC survival.^43^ Our interaction analysis and stratified analysis shows that smoker had a shorter survival after chemotherapy. The impact of alcohol on CRC is controversial. A recent study finds high alcohol consumption is dangerous for early‐onset CRC.^44^ Our research supported that smoker had worse prognosis than non‐smokers. Our results are consistent with the meta‐analysis that supported that men had worse survival.^45^ Notably, some studies have declared that androgen related to a lower risk of CRC in males.^46^, ^47^ The link between sex hormones and CRC needs more research.

The most ordinary secondary actions of the irinotecan‐based chemotherapy regimen are hematological and gastrointestinal toxicity.^48^ Studies have suggested that genetic variations could regulate cancer prognosis by affecting gene expression levels.^49^ In this study, we found irinotecan‐based chemotherapy was markedly correlated to decreased PFS and OS of patients with CC/CT genotype of rs4288573. In the stratified analysis of patients treated with irinotecan chemotherapy, we also found that smokers with CC/CT genotype of rs4288573 had lower OS and PFS. Perhaps smoking affects the enzyme that metabolizes irinotecan, thus reducing the effectiveness of irinotecan.^50^

There are also some limitations in our research. First of all, since we only verified our fundings in the TCGA and UKBB cohorts, and the association between rs4288573 and CRC prognosis needed to be further verified in another Chinese population cohort. Second, the biological function of the candidate SNPs needs further experimental verification. Finally, the mechanism of primary cilia affecting the survival of CRC needs more explorations.

Briefly, this study concentrates on the correlation between genetic variation of primary cilia‐related genes and CRC prognosis. Our findings revealed the importance of genetic variants of *ODF2L* and provided new insights into the prediction of chemotherapy resistance.

## AUTHOR CONTRIBUTIONS

**Lei Qiu:** Formal analysis (equal); writing – original draft (lead); writing – review and editing (equal). **Silu Chen:** Formal analysis (equal); writing – review and editing (equal). **Shuai Ben:** Data curation (equal); writing – review and editing (equal). **Jinxin Cui:** Investigation (equal); resources (equal). **Shan Lu:** Investigation (lead). **Rong Qu:** Investigation (equal). **Jinghuan Lv:** Funding acquisition (equal); validation (equal). **Wei Shao:** Validation (equal). **Qiang Yu:** Conceptualization (lead); funding acquisition (lead).

## FUNDING INFORMATION

Gusu Health Talents Program (GSWS2021040 and GSWS2019054) supported our study.

## CONFLICT OF INTEREST STATEMENT

The authors made no disclosures.

## ETHICS STATEMENT

The study was approved by the institution review board of Nanjing Medical University.

## Supporting information


Data S1:

## Data Availability

The data that support the findings of this study are available within the Supporting Information files and from the corresponding author upon reasonable request.
