# Supplementary material for: Genetic variants in primary cilia‐related genes associated with the prognosis of first‐line chemotherapy in colorectal cancer
Source: Cancer Med. 2024 Feb 9;13(2):e6996. doi: 10.1002/cam4.6996 (PMC10854446; doi:10.1002/cam4.6996)
Supplement: Supplementary file 1 — Data S1: [file CAM4-13-e6996-s001.docx]

**Supplementary materials and methods**

**Validation cohort of the UK biobank and TCGA**

We collected colorectal cancer patients from TCGA database with prognostic information as an independent cohort. The UK Biobank cohort was a prospective, population-based study that recruited adults aged 40-69 years from the general population between 2006 and 2010. The association between candidate SNPs and survival of colorectal cancer was further validated in the UK biobank and the TCGA cohorts.

**RNA extraction and quantitative real-time PCR analyses**

Total RNA from cells was extracted by TRIzol (Invitrogen) regent according to the Manufacturer’s protocol. Then, total RNA samples were detected by ultraviolet spectrometry and was converted to cDNA with PrimeScript^TM^ RT Master Mix (TaKara). Quantitative real-time PCR (qRT-PCR) was performed on a Roche LightCycler 480 PCR System with AceO qPCR SYBR Green Master Mix (Vazyme). The cNDA samples were evaluated by triplicate. The PCR conditions on the following cycle: 95℃ for 5 min, followed by 45 cycles of 95℃ for 15s and 60℃ for 60s. The mRNA expression was normalized against the endogenous expression of GAPDH. The primer sequences were shown in **Supplementary Table 6**.

**Protein extraction and western blot**

Cells were lysed in RIPA lysis buffer (Beyotime) supplemented with the protease inhibitor PMSF (Beyotime). The protein concentration was quantified by a BCA protein assay kit (Beyotime). The lysates were combined with 6 × SDS-PAGE sample buffer and β-mercaptoethanol and denatured at 100°C for 5 min. Equal amounts of each protein sample were separated by 10% SDS–polyacrylamide gel electrophoresis and transferred to a 0.22μm PVDF membrane (Millipore). The membrane was blocked with 5% non-fat dried milk at room temperature for 2h. After blocking, the membranes were incubated with anti-ODF2L (1:1500, Proteintech) or anti-GAPDH (1:20000, Proteintech) antibody. Immunoreactive proteins were visualized using a molecular imager (Bio-Rad).

**Cell lines and culture**

Colorectal cancer cell lines were obtained from shanghai Institute of Biochemistry and Cell Biology, and Chinese Academy of Science (Shanghai, China) (HT-29, HCT116, SW480, DLD-1, Caco-2 and Lovo). SW480, DLD-1, Lovo and Caco-2 cell lines were maintained in DMED High Glucose (Bionid), HT-29, HCT116 and were cultured in PRMI 1640 (Bionid). All media were supplemented with 10% fetal bovine serum (Bionid) and 100U/ml penicillin and streptomycin. The cells were cultured at 37℃ in a humidified atmosphere of 5% CO_2_.

**Cell transfection and cell proliferation**

To knock down ODF2L in colon cell lines, the ODF2L siRNA was designed. Lipofectamine 2000 transfection reagent **(**Invitrogrn) was used according to instructions. HCT-116 and DLD-1 cell proliferation was analyzed buy a Cell Counting Kit-8 (CCK-8; Dojindo). Cells were collected and reseeded into a 96-well plate and cultured. Next, a mixture of 10uL of CCK-8 solution and 100uL of medium was added to each well and incubated at room temperature for 2h. The absorbance was measured at 450nm on a plate reader (Bio Tek). All experiments were conducted in triplicate.

**Cell viability**

Cell viability was measured by SRB assays. Briefly, cells were seeded in 96-well plates and replaced with conditioned medium after adherence. Then, cells were cultured for the indicated time and fixed with 10% trichloroacetic acid at room temperature for 30 min, followed by incubation with 0.4% SRB (w/v) solution in 1% acetic acid for 20 min at room temperature. Finally, the SRB was dissolved with 10mM unbuffered Tris base and the absorbance was measured at a wavelength of 530nm on a plate reader (Bio Tek).

**URLs**

HaploReg v4.1: https://pubs.broadinstitute.org/mammals/haploreg/haploreg.php

RegulomDB: https://regulome.stanford.edu/regulome-search/

3D SNP: https://www.omic.tech/3dsnpv2/

PPI: https://cn.string-db.org/

RNA fold: http://rna.tbi.univie.ac.at/cgi-bin/RNAWebSuite/RNAfold.cgi

TISIDB database: http://cis.hku.hk/TISIDB/index.php

GTEx: https://www.gtexportal.org/

CancerSplicingQTL database: http://www.cancersplicingqtl-hust.com/#/

TIMER database: https://cistrome.shinyapps.io/timer/

**Supplementary Figure legends**

**Supplementary Figure 1. The interaction of 27 key genes in primary cilia.** (A) The fold change of 27 primary-cilia genes expression in tumor tissues and normal tissues in the TCGA database. (B) Protein-protein interaction (PPI) network among 27 primary-related genes.

**Supplementary Figure 2. Kaplan-Meier curve of rs4288573 in the UK biobank cohort.** (A) Kaplan Meier curves of overall survival (OS) for rs4288573 in colorectal cancer. (B) Kaplan Meier curves of cancer-specific survival (CSS) for rs4288573 in colorectal cancer.

**Supplementary Figure 3. The interaction effects between rs4288573 and smoking status on colorectal cancer overall survival (OS), HR adjusted for age and sex.**

**Supplementary Figure 4. The association between rs4288573 and progression-free survival (PFS) and overall-survival (OS) of patients with oxaliplatin-based chemotherapy in four genetic models.**

**Supplementary Figure 5. Stratified analysis of the association between rs4288573 and disease control rate (DCR) of colorectal cancer patients treated with irinotecan-based chemotherapy under the dominant model.**

**Supplementary Figure 6. Expression analysis of *ODF2L* in TCGA database.** (A) Expression of *ODF2L* in colorectal cancer tissues stratified by age. (B) Expression of *ODF2L* in colorectal cancer tissues stratified by BMI. (C) Expression of *ODF2L* in colorectal cancer tissues stratified by sex. (D) Expression of *ODF2L* in colorectal cancer tissues stratified by tumor site.

**Supplementary Figure 7. Correlation of *ODF2L* expression with immune infiltration in colorectal cancer.** (A) Correlation between *ODF2L* expression and the abundance of tumor-infiltrating immune cells in colorectal cancer available from the TIMER2.0 database. (B) The infiltration levels of various immune cells under different copy numbers of *ODF2L*. COAD colon adenocarcinoma; READ rectal adenocarcinoma.

**Supplementary Figure 8. The expression of *ODF2L* is associated with immunomodulators in colorectal cancer.** (A) Correlation between ODF2L expression and immunostimulators in colorectal cancer available at TISIDB database. (B) Correlation between *ODF2L* expression and immune inhibitors in colorectal cancer available at TISIDB database.

**Supplementary Figure 9. Correlation between *ODF2L* expression and chemokines in colorectal cancer at TISIDB database.**

**Supplementary Figure 10. Expression of ODF2L in colon cancer cell lines.** (A) The mRNA expression level of ODF2L in six colon cancer cell lines. (B) The knockdown of ODF2L protein expression in HCT-116 and DLD-1 cells by ODF2L siRNA were determined by western blot.

**Supplementary Figure 11. *ODF2L* regulate proliferation and chemotherapy resistance in colon cancer cells.** (A, B) *ODF2L* knockdown by transient siRNA transfection suppressed the proliferation of HCT-116 and DLD-1 cells. (C, D) Effect of knockdown ODF2L increased sensitivity of colon cancer cells to irinotecan. HCT-116 and DLD-1 cells were cultured with conditioned medium containing 50μM and 100μM irinotecan for 2-5 days.

**Supplementary Figure 1.
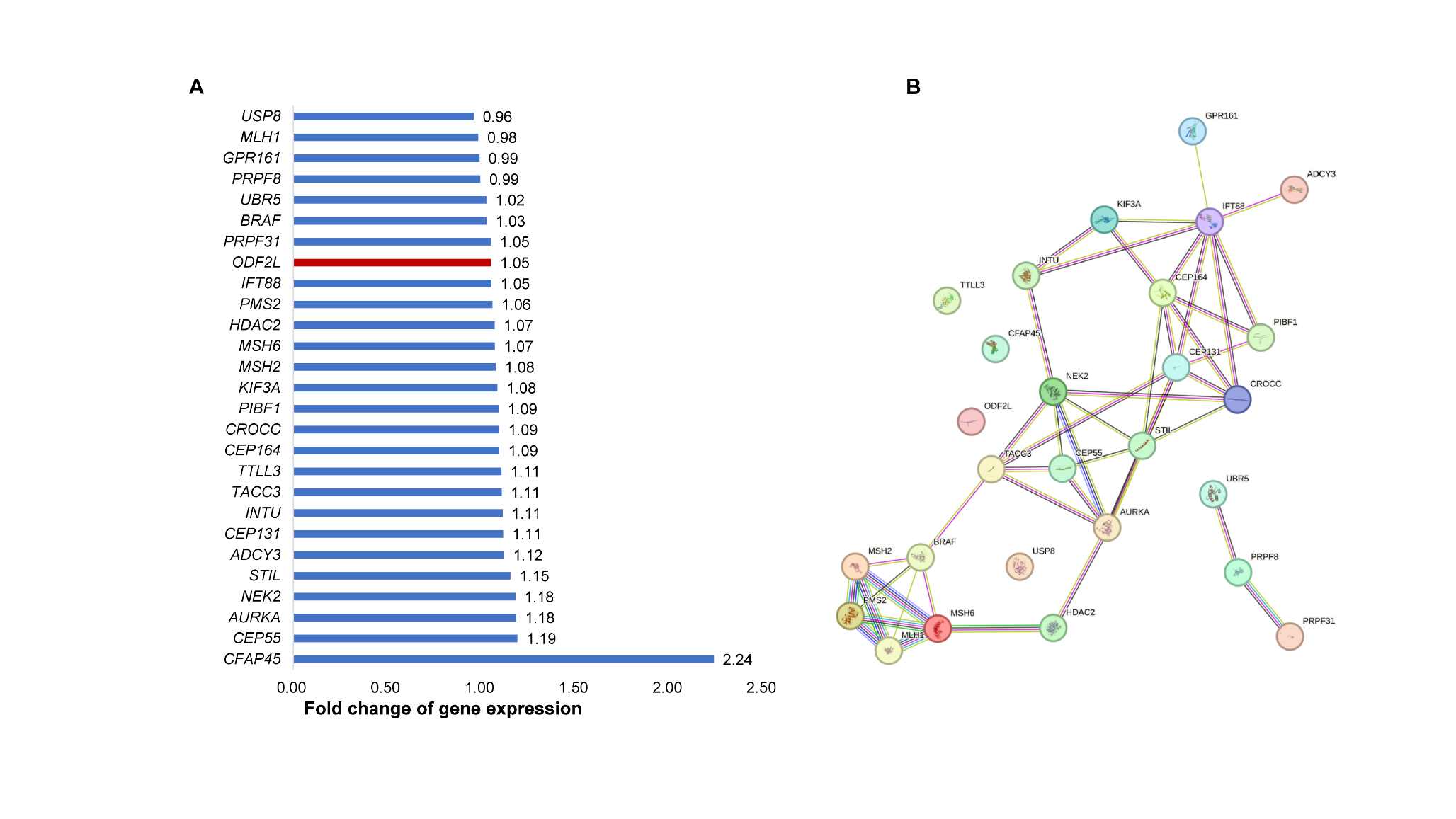
**

**
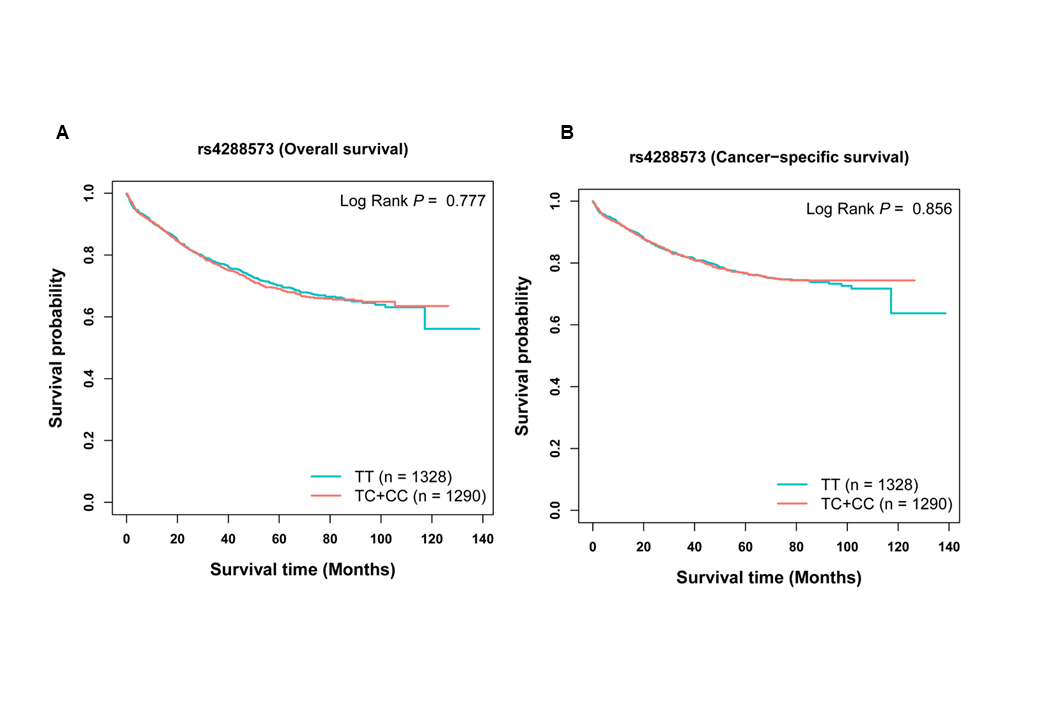
Supplementary Figure 2.**

**
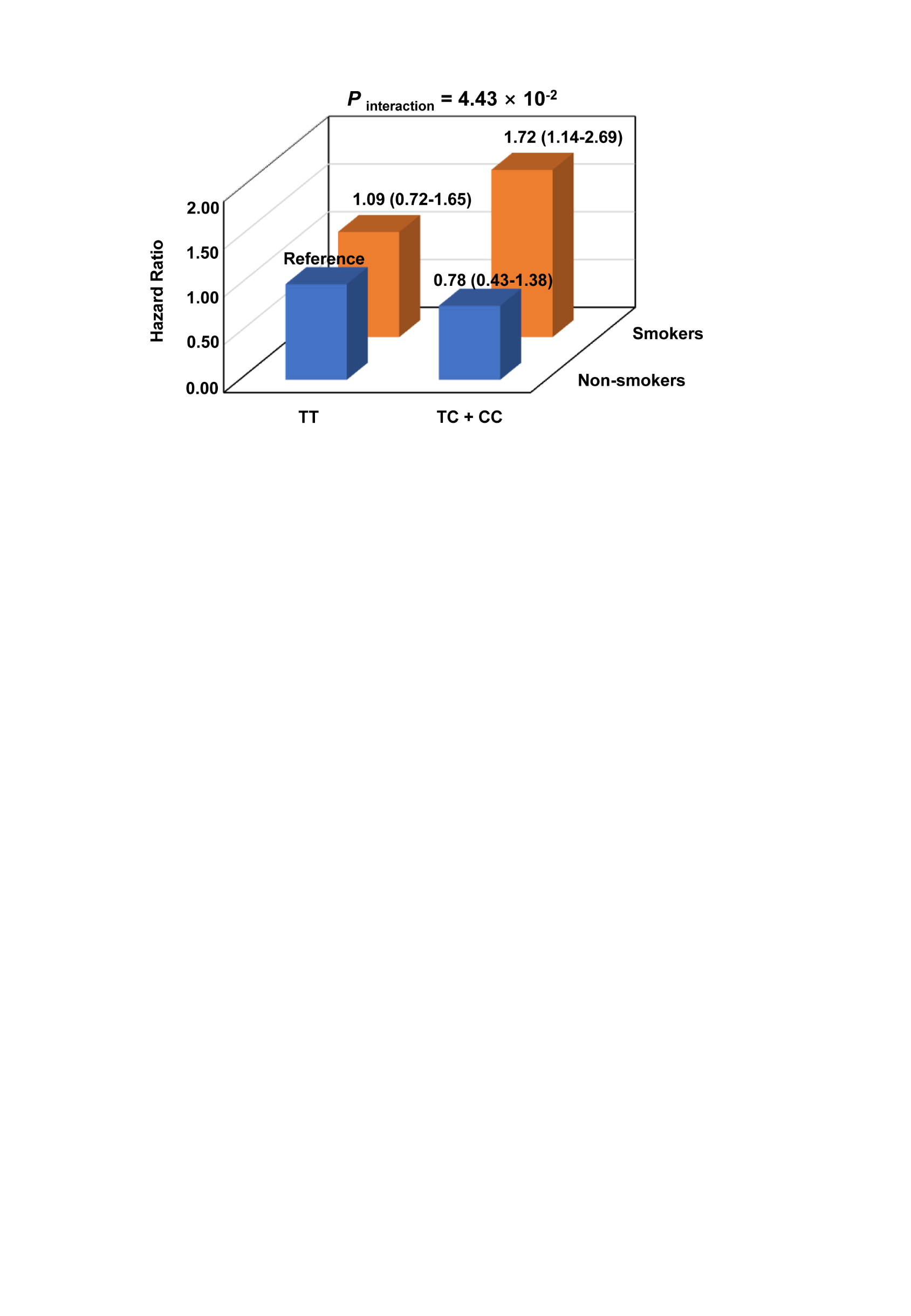
Supplementary Figure 3.**

**Supplementary Figure 4.**

**
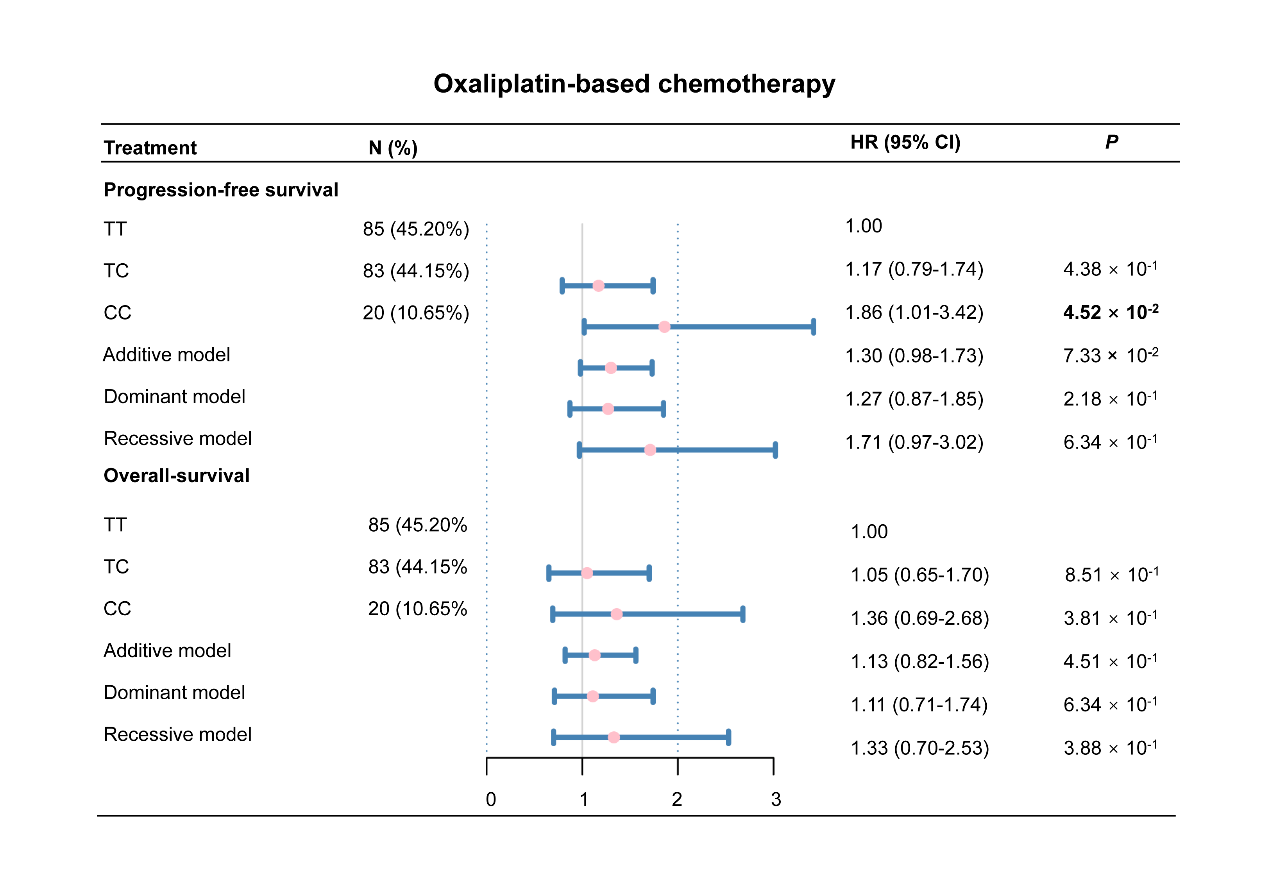
**

**Supplementary Figure 5.**

**
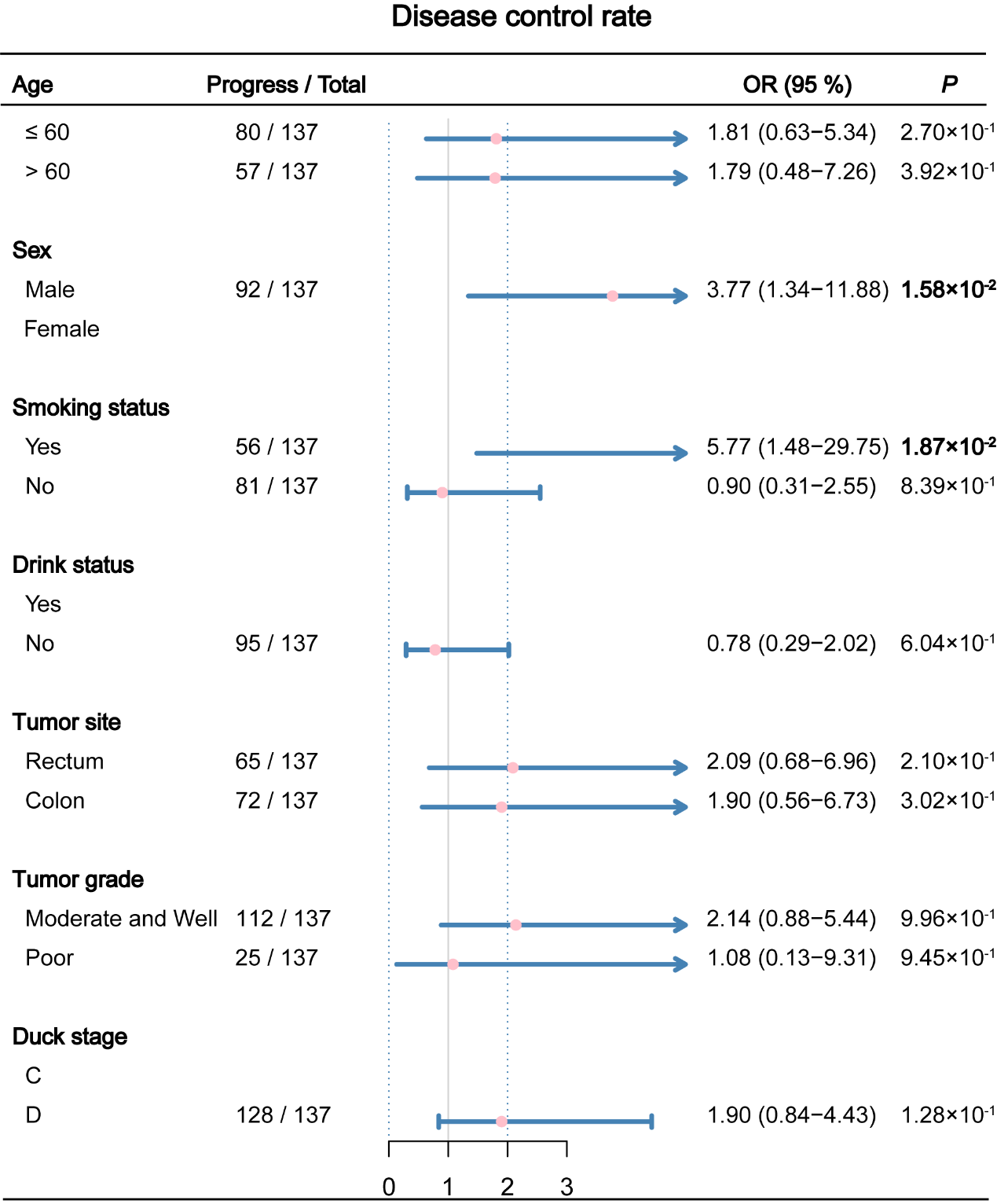
**

**Supplementary Figure 6.
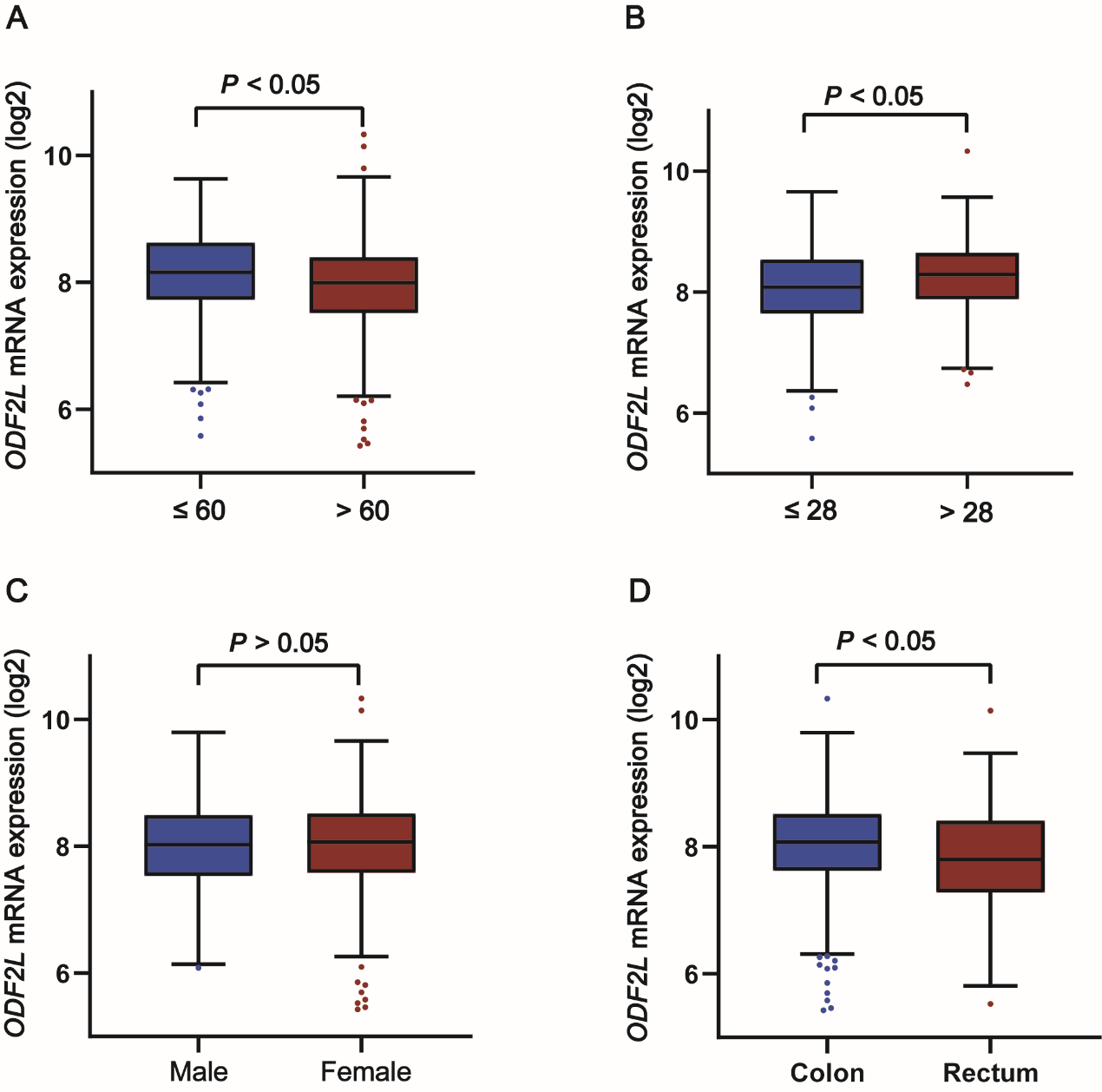
**

**Supplementary Figure 7.**

**
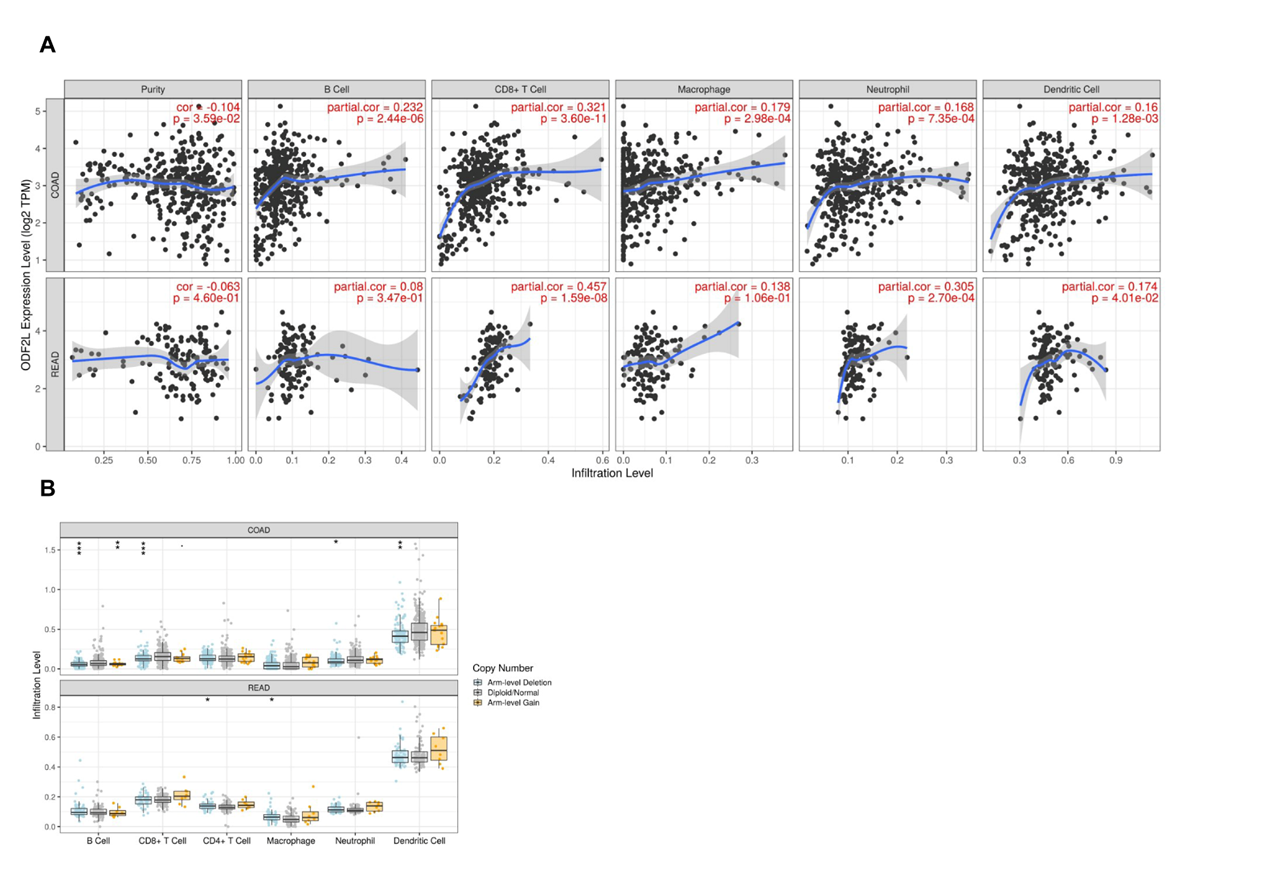
**

**Supplementary Figure 8.**


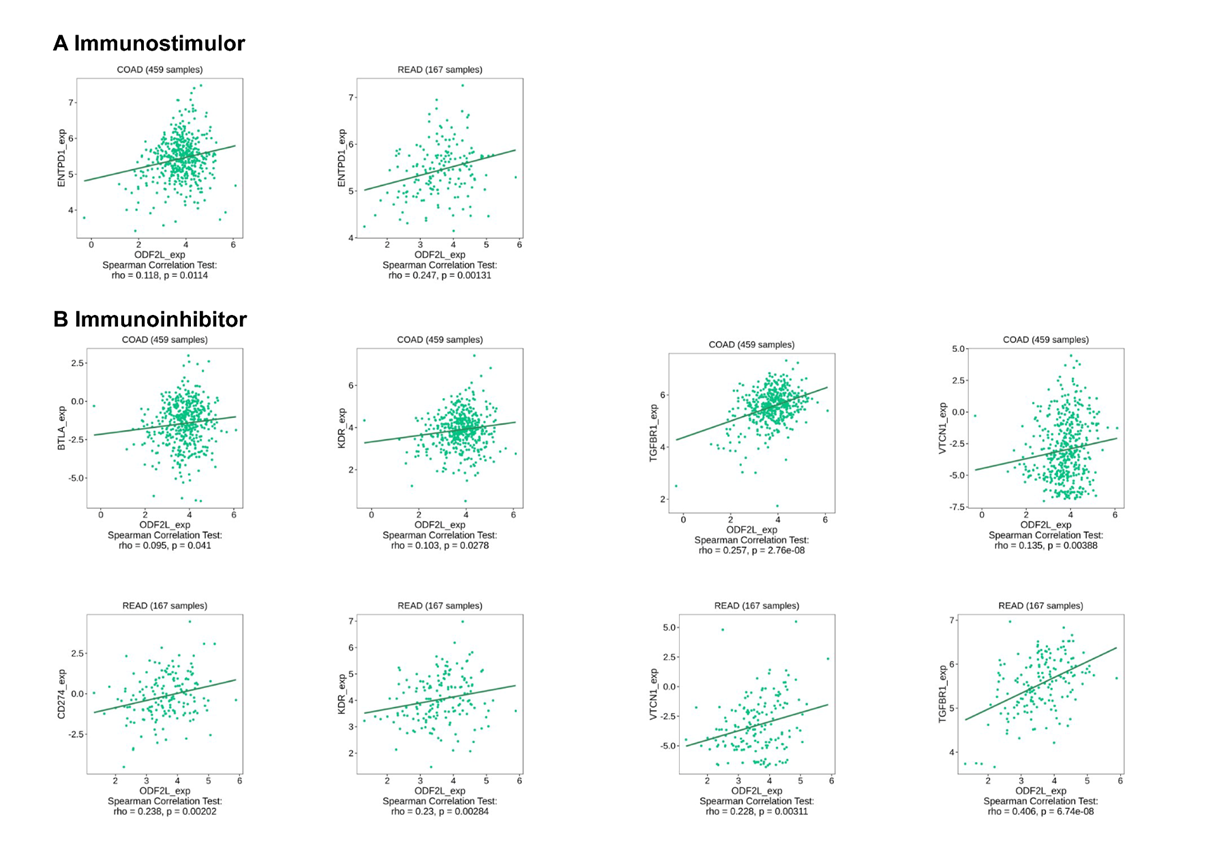


**
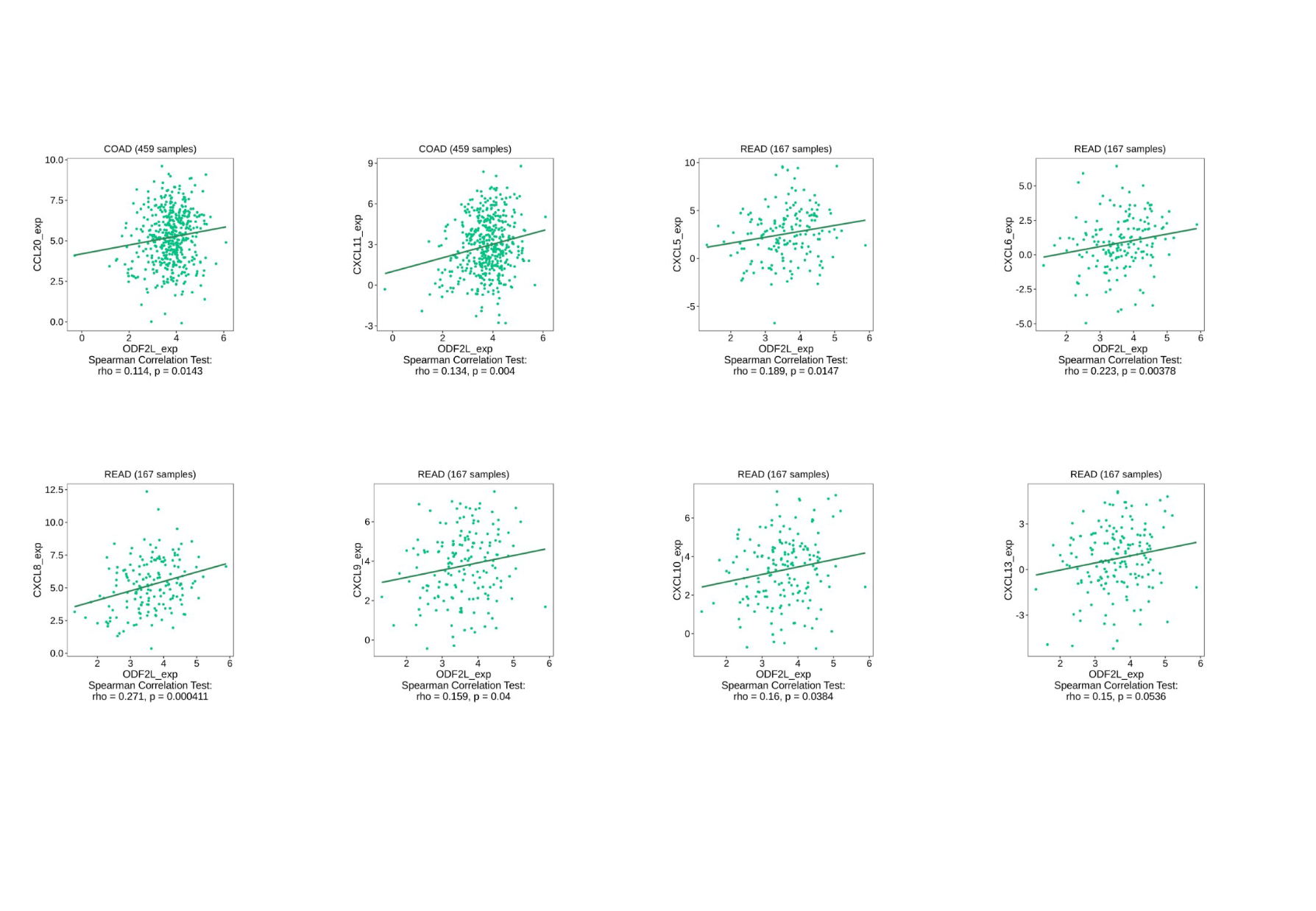
Supplementary Figure 9.**

**Supplementary Figure 10.**

**
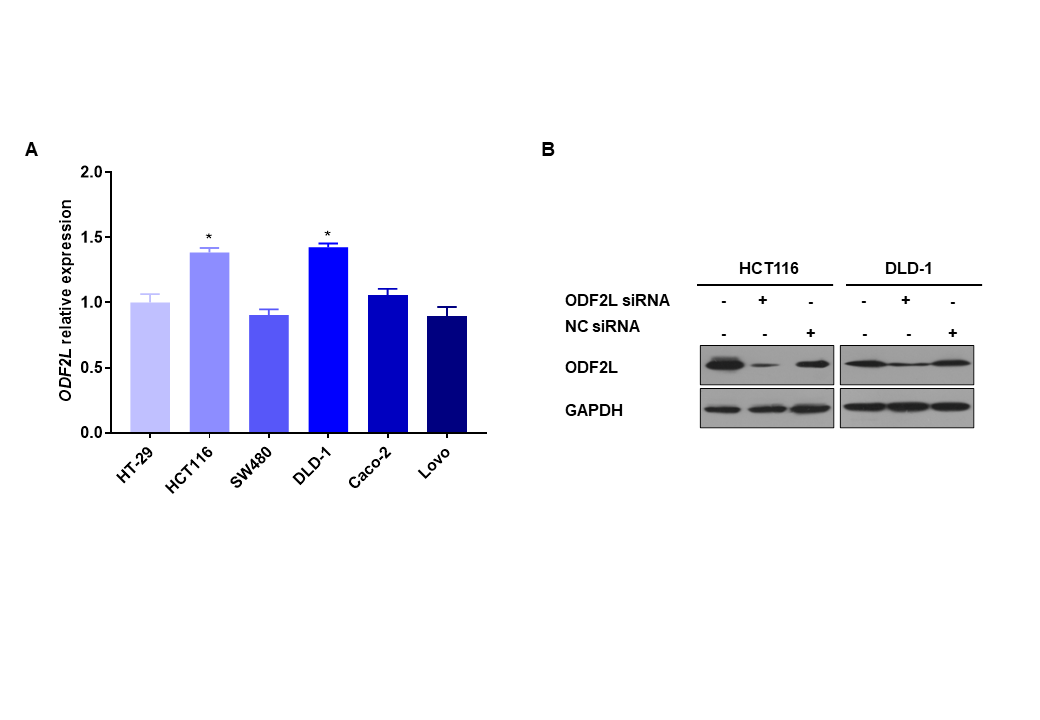
**

**Supplementary Figure 11.**

**
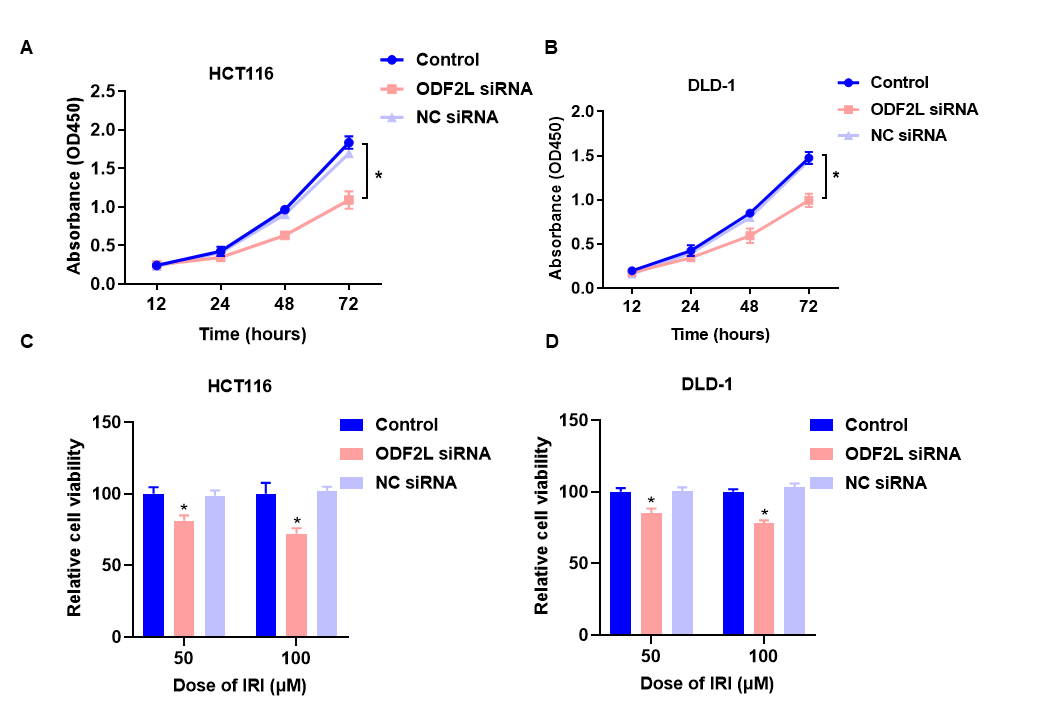
**

**Supplementary Table 1. The key genes in the primary-cilia-related genes.**

| **Gene** | **Chr** | **Position (hg 19) ^a^** | **Functional description** | **First author** | **Year** |
| --- | --- | --- | --- | --- | --- |
| *NEK2* | 1 | 211831598-211848963 | Inhibiting primary cilia formation | Sehyun Kim *et al.* | 2015 |
| *CFAP45* | 1 | 159842154-159869955 | A component essential for primary cilium formation | Gerard W Dougherty *et al.* | 2020 |
| *GPR161* | 1 | 168048780-168106905 | A component essential for primary cilium formation | Elena A May *et al.* | 2021 |
| *ODF2L* | 1 | 86812520-86862006 | Inhibiting primary cilia formation | Paul de Saram *et al.* | 2017 |
| *STIL* | 1 | 47715811-47780568 | Related to centrosome | Jingxian Li et al. | 2023 |
| *CROCC* | 1 | 17248426-17299459 | Related to centrosome | Andrea Remo et al. | 2018 |
| *ADCY3* | 2 | 25042038-25143106 | A component essential for primary cilium formation | Acqueline E Siljee et al. | 2018 |
| *MSH2* | 2 | 47630206- 47710367 | Mismatch repair gene | Antonio Russo et al. | 2009 |
| *MSH6* | 2 | 48010284- 48034092 | Mismatch repair gene | Nargisse Nejda et al. | 2009 |
| *TTLL3* | 3 | 9851411-9878049 | Inhibiting primary cilia formation | Cecilia Rocha et al. | 2014 |
| *MLH1* | 3 | 37035009-37092337 | Mismatch repair gene | Jeanne Perrin et al. | 2001 |
| *TACC3* | 4 | 1723248-1746898 | Inhibiting primary cilia formation | Yunkai Qie et al. | 2020 |
| *INTU* | 4 | 128554112-128647892 | A component essential for primary cilium formation | N Yang et al. | 2017 |
| *KIF3A* | 5 | 132028323-132073238 | A component essential for primary cilium formation | Carlie L Cullen et al. | 2021 |
| *HDAC2* | 6 | 114254192-114292312 | A component essential for primary cilium formation | Tetsuo Kobayashi et al. | 2017 |
| *PMS2* | 7 | 6010556- 6048737 | Mismatch repair gene | Alexandra Khichfy Alex et al. | 2016 |
| *BRAF* | 7 | 140413128- 140624729 | BRAF mutation CRC | E Sanz-Garcia et al. | 2017 |
| *UBR5* | 8 | 103264501-103424928 | A component essential for primary cilium formation | Robert F Shearer *et al.* | 2018 |
| *CEP55* | 10 | 95256369-95288849 | Promotes the breakdown of primary cilia | Yu-Cheng Zhang *et al.* | 2021 |
| *CEP164* | 11 | 117192494-117283982 | A component essential for primary cilium formation | Susanne Graser *et al.* | 2007 |
| *PIBF1* | 13 | 73356271-73590599 | A component essential for primary cilium formation | Kyeongmi Kim *et al.* | 2012 |
| *IFT88* | 13 | 21141296-21265583 | A component essential for primary cilium formation | Jonathan M Scholey *et al.* | 2003 |
| *USP8* | 15 | 50716602-50806618 | A component essential for primary cilium formation | Albino Troilo *et al.* | 2014 |
| *PRPF8* | 17 | 1553923-1588161 | A component essential for primary cilium formation | Gabrielle Wheway *et al.* | 2015 |
| *CEP131* | 17 | 79163396-79196765 | A component essential for primary cilium formation | Christopher J Staples *et al.* | 2012 |
| *PRPF31* | 19 | 54619134-54635144 | A component essential for primary cilium formation | Gabrielle Wheway et al. | 2015 |
| *AURKA* | 20 | 54944446- 54967271 | Related to centrosome | Noushin Miralaei et al. | 2021 |

^a^ Based on NCBI build 19 of the human genomes.

**Supplementary Table 2.** **The association of** **28 SNPs with the survival of colorectal cancer.**

| **CHR** | **SNP** | **Position (hg19)** | **Allele ^a^** | **MAF** | **Gene** | **Location** | **PFS** | | |  | **OS** | | |
| --- | --- | --- | --- | --- | --- | --- | --- | --- | --- | --- | --- | --- | --- |
|  |  |  |  |  |  |  | **HR (95% CI) ^b^** | ***P*** | ***P*_FDR_** |  | **HR (95% CI) ^b^** | ***P*^c^** | ***P*_FDR_** |
| 1 | rs4288573 | 86861087 | T > C | 0.327 | *ODF2L* | Intronic | 1.39 (1.14-1.70) | **1.36 × 10^-3^** | **3.82 × 10^-2^** |  | 1.31 (1.03-1.65) | **2.62 × 10^-2^** | 3.67 × 10^-1^ |
| 1 | rs35711761 | 47778833 | T > C | 0.192 | *STIL* | Intronic | 0.72 (0.56-0.92) | **8.91 × 10^-3^** | 1.25 × 10^-1^ |  | 1.03 (0.78-1.37) | 8.37 × 10^-1^ | 9.80 × 10^-1^ |
| 1 | rs3125632 | 47779951 | A > G | 0.435 | *STIL* | 5'-UTR | 0.80 (0.66-0.96) | **1.87 × 10^-2^** | 1.75 × 10^-1^ |  | 1.11 (0.88-1.40) | 3.59 × 10^-1^ | 9.80 × 10^-1^ |
| 1 | rs6683267 | 86859252 | C > T | 0.488 | *ODF2L* | Intronic | 0.83 (0.69-1.00) | **4.43 × 10^-2^** | 3.10 × 10^-1^ |  | 1.00 (0.80-1.25) | 9.80 × 10^-1^ | 9.80 × 10^-1^ |
| 2 | rs2303425 | 47630213 | T > C | 0.175 | *MSH2* | 5'-UTR | 1.26 (0.99-1.61) | 6.51 × 10^-2^ | 3.64 × 10^-1^ |  | 0.93 (0.68-1.26) | 6.32 × 10^-1^ | 9.80 × 10^-1^ |
| 1 | rs12097163 | 17293660 | C > G | 0.111 | *CROCC* | Intronic | 0.81 (0.61-1.08) | 1.44 × 10^-1^ | 6.73 × 10^-1^ |  | 0.90 (0.61-1.32) | 5.89 × 10^-1^ | 9.80 × 10^-1^ |
| 1 | rs2273112 | 17287639 | C > T | 0.088 | *CROCC* | Intronic | 0.83 (0.61-1.13) | 2.33 × 10^-1^ | 8.42 × 10^-1^ |  | 0.84 (0.55-1.28) | 4.23 × 10^-1^ | 9.80 × 10^-1^ |
| 4 | rs2282763 | 1728120 | A > C | 0.168 | *TACC3* | Intronic | 0.87 (0.67-1.11) | 2.61 × 10^-1^ | 8.42 × 10^-1^ |  | 0.90 (0.66-1.24) | 5.32 × 10^-1^ | 9.80 × 10^-1^ |
| 1 | rs11586522 | 168091031 | C > A | 0.197 | *GPR161* | Intronic | 1.13 (0.89-1.44) | 3.02 × 10^-1^ | 8.42 × 10^-1^ |  | 1.05 (0.78-1.40) | 7.66 × 10^-1^ | 9.80 × 10 |
| 17 | rs9911096 | 79196662 | C > G | 0.150 | *CEP131* | 5'-UTR | 0.88 (0.68-1.14) | 3.27 × 10^-1^ | 8.42 × 10^-1^ |  | 1.03 (0.76-1.41) | 8.47 × 10^-1^ | 9.80 × 10 |
| 15 | rs2241769 | 50716633 | G > C | 0.487 | *USP8* | 5'-UTR | 0.91 (0.75-1.10) | 3.31 × 10^-1^ | 8.42 × 10^-1^ |  | 0.80 (0.64-1.01) | 6.54 × 10^-2^ | 6.10 × 10^-1^ |
| 1 | rs2501320 | 159860259 | G > A | 0.188 | *CFAP45* | Intronic | 0.90 (0.71-1.13) | 3.69 × 10^-1^ | 8.61 × 10^-1^ |  | 0.98 (0.74-1.29) | 8.78 × 10^-1^ | 9.80 × 10 |
| 1 | rs1182099 | 86821974 | A > T | 0.186 | *ODF2* | Intronic | 0.90 (0.71-1.16) | 4.20 × 10^-1^ | 9.05 × 10^-1^ |  | 0.65 (0.47-0.89) | **7.74 × 10^-3^** | 2.17 × 10^-1^ |
| 8 | rs6468826 | 103420337 | G > C | 0.492 | *UBR5* | Intronic | 0.93 (0.76-1.14) | 4.76 × 10^-1^ | 9.14 × 10^-1^ |  | 0.85 (0.67-1.07) | 1.67 × 10^-1^ | 9.37 × 10^-1^ |
| 19 | rs4806711 | 54619191 | A > G | 0.215 | *PRPF31* | Intronic | 0.92 (0.73-1.17) | 5.09 × 10^-1^ | 9.14 × 10^-1^ |  | 1.06 (0.82-1.37) | 6.58 × 10^-1^ | 9.80 × 10^-1^ |
| 1 | rs16828104 | 168090899 | C > T | 0.245 | *GPR161* | Intronic | 1.07 (0.86-1.35) | 5.42 × 10^-1^ | 9.14 × 10^-1^ |  | 0.97 (0.73-1.29) | 8.37 × 10^-1^ | 9.80 × 10^-1^ |
| 1 | rs2501327 | 159852240 | T > A | 0.201 | *CFAP45* | Intronic | 0.93 (0.74-1.17) | 5.57 × 10^-1^ | 9.14 × 10^-1^ |  | 0.99 (0.76-1.30) | 9.63 × 10^-1^ | 9.80 × 10^-1^ |
| 3 | rs3774343 | 37035834 | A > G | 0.052 | *MLH1* | Intronic | 0.89 (0.59-1.35) | 5.88 × 10^-1^ | 9.14 × 10^-1^ |  | 0.99 (0.59-1.67) | 9.65 × 10^-1^ | 9.80 × 10^-1^ |
| 17 | rs969413 | 79195814 | A > T | 0.317 | *CEP131* | Intronic | 1.04 (0.85-1.28) | 6.82 × 10^-1^ | 9.24 × 10^-1^ |  | 1.10 (0.86-1.41) | 4.42 × 10^-1^ | 9.80 × 10^-1^ |
| 2 | rs7567997 | 25096952 | T > C | 0.497 | *ADCY3* | Intronic | 0.97 (0.80-1.18) | 7.49 × 10^-1^ | 9.24 × 10^-1^ |  | 1.09 (0.86-1.37) | 4.84 × 10^-1^ | 9.80 × 10^-1^ |
| 7 | rs2286681 | 6035428 | A > C | 0.426 | *PMS2* | Intronic | 1.03 (0.85-1.24) | 7.71 × 10^-1^ | 9.24 × 10^-1^ |  | 0.97 (0.78-1.22) | 8.12 × 10^-1^ | 9.80 × 10^-1^ |
| 4 | rs3134866 | 1723265 | G > A | 0.136 | *TACC3* | 5'-UTR | 0.96 (0.74-1.26) | 7.71 × 10^-1^ | 9.24 × 10^-1^ |  | 1.01 (0.73-1.40) | 9.69 × 10^-1^ | 9.80 × 10^-1^ |
| 15 | rs28366846 | 50718969 | C > G | 0.076 | *USP8* | Intronic | 0.96 (0.66-1.38) | 8.20 × 10^-1^ | 9.24 × 10^-1^ |  | 1.07 (0.68-1.68) | 7.78 × 10^-1^ | 9.80 × 10^-1^ |
| 1 | rs4656855 | 159859260 | T > C | 0.435 | *CFAP45* | Intronic | 0.98 (0.81-1.18) | 8.25 × 10^-1^ | 9.24 × 10^-1^ |  | 1.06 (0.85-1.31) | 6.13 × 10^-1^ | 9.80 × 10^-1^ |
| 3 | rs11717031 | 9869150 | G > T | 0.178 | *TTLL3* | Intronic | 0.98 (0.76-1.26) | 8.73 × 10^-1^ | 9.24 × 10^-1^ |  | 0.97 (0.71-1.34) | 8.59 × 10^-1^ | 9.80 × 10^-1^ |
| 5 | rs2277065 | 132073002 | G > A | 0.244 | *KIF3A* | Intronic | 0.98 (0.79-1.23) | 8.79 × 10^-1^ | 9.24 × 10^-1^ |  | 0.81 (0.61-1.09) | 1.67 × 10^-1^ | 9.37 × 10^-1^ |
| 2 | rs6545790 | 25109302 | A > G | 0.439 | *ADCY3* | Intronic | 1.01 (0.84-1.22) | 8.91 × 10^-1^ | 9.24 × 10^-1^ |  | 1.12 (0.89-1.41) | 3.33 × 10^-1^ | 9.80 × 10^-1^ |
| 2 | rs2303426 | 47630550 | G > C | 0.190 | *MSH2* | Intronic | 1.00 (0.79-1.27) | 9.97 × 10^-1^ | 9.97 × 10^-1^ |  | 1.20 (0.90-1.59) | 2.21 × 10^-1^ | 9.80 × 10^-1^ |

^a^ Reference allele/effect allele.

^b^ Adjusted for age, sex, smoking status, and drinking status.

Abbreviation: MAF, minor allele frequency; PFS, progression‐free survival; OS, overall survival; HR: hazard ratio; CI: confidence interval;

*P*_FDR_, after false discovery rate correction.

**Supplementary Table 3. Association between rs4288573 and survival of colorectal cancer patients in four genetic models.**

| **Model** | **N (%)** | **OS** | |
| --- | --- | --- | --- |
|  |  | **HR (95% CI) ^a^** | ***P*^a^** |
| TT | 216 (42.52%) | 1.00 |  |
| TC | 234 (46.06%) | 0.78 (0.52-1.17) | 2.22 × 10^-1^ |
| CC | 58 (11.42%) | 0.66 (0.32-1.35) | 2.51 ×10^-1^ |
| Additive model |  | 0.80 (0.59-1.08) | 1.44 ×10^-1^ |
| Dominant model |  | 0.75 (0.51-1.11) | 1.56 ×10^-1^ |
| Recessive model |  | 0.75 (0.38-1.49) | 4.13 ×10^-1^ |

^a^ Adjusted for sex, and age in the Cox regression model.

OS: overall survival; HR: hazard ratio; CI: confidence interval.

**Supplementary Table 4. Stratified analysis of the association between rs4288573 and survival of colorectal cancer patients receiving irinotecan-based chemotherapy in the dominant model.**

|  | **PFS** | |  | **OS** | |
| --- | --- | --- | --- | --- | --- |
| **Variables** | **HR (95% CI) ^a^** | ***P*^a^** |  | **HR (95% CI) ^a^** | ***P*^a^** |
| **Age** |  |  |  |  |  |
| ≤60 | 1.54 (0.89-2.66) | 1.24 × 10^-1^ |  | 3.44 (1.63-7.28) | **1.24 × 10^-3^** |
| >60 | 2.73 (1.34-5.56) | **5.48 × 10^-3^** |  | 0.93 (0.43-2.01) | 8.56 × 10^-1^ |
| **Sex** |  |  |  |  |  |
| Male | 2.00 (1.24-3.24) | **4.57 × 10^-3^** |  | 3.37 (1.74-6.54) | **3.18 × 10^-4^** |
| Female | 1.08 (0.48-2.41) | 8.57 × 10^-1^ |  | 0.98 (0.38-2.54) | 9.69 × 10^-1^ |
| **Smoking Status** |  |  |  |  |  |
| Yes | 2.67 (1.37-5.21) | **4.07 × 10^-3^** |  | 3.27 (1.36-7.87) | **8.12 × 10^-3^** |
| No | 1.28 (0.76-2.17) | 3.57 × 10^-1^ |  | 1.42 (0.73-2.75) | 2.99 × 10^-1^ |
| **Drinking Status** |  |  |  |  |  |
| Yes | 3.20 (1.43-7.16) | **4.60 × 10^-3^** |  | 2.23 (0.80-6.21) | 1.25 × 10^-1^ |
| No | 1.35 (0.84-2.19) | 2.18 × 10^-1^ |  | 1.98 (1.07-3.66) | **2.93 × 10^-2^** |
| **Tumor site** |  |  |  |  |  |
| Rectum | 3.16 (1.62-6.18) | **7.43 × 10^-4^** |  | 2.30 (1.11-4.74) | **2.44 × 10^-2^** |
| Colon | 1.55 (0.87-2.75) | 1.38 × 10^-1^ |  | 1.73 (0.81-3.70) | 1.55 × 10^-1^ |
| **Tumor grade** |  |  |  |  |  |
| Moderate and well | 2.03 (1.29-3.21) | **2.29 × 10^-3^** |  | 2.43 (1.34-4.38) | **3.35 × 10^-3^** |
| Poor | 2.65 (0.75-9.32) | 1.28 × 10^-1^ |  | 2.53 (0.71-9.02) | 1.51 × 10^-1^ |
| **Dukes stage** |  |  |  |  |  |
| C | 0.73 (0.06-8.71) | 8.04 × 10^-1^ |  | 2.25 (0.14-36.79) | 5.69 × 10^-1^ |
| D | 1.76 (1.16-2.67) | **7.64 × 10^-3^** |  | 2.24 (1.32-3.79) | **2.86 × 10^-3^** |
| **Metastasis** |  |  |  |  |  |
| ≤2 | 2.06 (1.26-3.39) | **3.92 × 10^-3^** |  | 2.37 (1.28-4.42) | **6.34 × 10^-3^** |
| >2 | 2.66 (0.65-10.86) | 1.73 × 10^-1^ |  | 2.52 (0.62-10.16) | 1.95 × 10^-1^ |

^a^ Adjusted for sex, age, smoking status, and drinking status in the Cox regression model.

PFS: progression-free survival; OS: overall survival.

**Supplementary Table 5. In silico analysis of functional annotation for 28 SNPs**

| **SNP** | **Gene** | **Regulome DB Score^a^** | **3D SNP^b^** | **HaploReg** |
| --- | --- | --- | --- | --- |
| rs4288573 | *ODF2L* | 1f | 122.98 | Promote histone marks, Enhancer histone marks, DNAse, Proteins bound, Motifs changed, Selected eQTL hits |
| rs35711761 | *STIL* | 1b | 80.00 | Promote histone marks, Enhancer histone marks, Proteins bound, Motifs changed, Selected eQTL hits |
| rs3125632 | *STIL* | 1a | 203.85 | SiPhy cons, Promoter histone marks, DNAse, Proteins bound, Motifs changed, Selected eQTL hits |
| rs6683267 | *ODF2L* | 1f | 18.18 | Promote histone marks, Enhancer histone marks, DNAse, Proteins bound, Selected eQTL hits |
| rs2303425 | *MSH2* | 1a | 203.49 | SiPhy cons, Promoter histone marks, DNAse, Proteins bound, Motifs changed, Selected eQTL hits |
| rs12097163 | *CROCC* | 1a | 27.70 | Promoter histone marks, Enhancer histone marks, DNAse, Proteins bound, Motifs changed, Selected eQTL hits |
| rs2273112 | *CROCC* | 1f | 127.72 | Promoter histone marks, Enhancer histone marks, DNAse, Proteins bound, Motifs changed, Selected eQTL hits |
| rs2282763 | *TACC3* | 1f | 180.49 | Promoter histone marks, Enhancer histone marks, DNAse, Proteins bound, Motifs changed, GRASP QTL hits, Selected eQTL hits |
| rs11586522 | *GPR161* | 1f | 26.07 | Promoter histone marks, Enhancer histone marks, DNAse, Proteins bound, Motifs changed, GRASP QTL Hits, Selected eQTL hits |
| rs9911096 | *CEP131* | 1f | 160.16 | Promoter histone marks, Enhancer histone marks, DNAse, Proteins bound, Motifs changed, Selected eQTL hits |
| rs2241769 | *USP8* | 1f | 202.46 | Promoter histone marks, DNAse, Proteins bound, Motifs changed, Selected eQTL hits |
| rs2501320 | *CFAP45* | 1f | 57.77 | Promoter histone marks, Enhancer histone marks, DNAse, Motifs changed, Selected eQTL hits |
| rs1182099 | *ODF2* | 1f | 33.08 | Enhancer histone marks, DNAse, Proteins bound, Motifs Changed, GRASP QTL hits, Selected eQTL hits |
| rs6468826 | *UBR5* | 1f | 121.63 | Promoter histone marks, Enhancer histone marks, DNAse, Proteins bound, Selected eQTL hits |
| rs4806711 | *UBR5* | 1b | 201.62 | Promoter Histone marks, DNAse, Proteins bound, Motifs changed, GRASP QTL hits, Selected eQTL hits |
| rs16828104 | *GPR161* | 1f | 20.90 | Promoter histone marks, Enhancer histone marks, DNAse, GRASP QTL hits, Selected eQTL hits |
| rs2501327 | *CFAP45* | 1f | 35.74 | Promoter histone marks, Enhancer histone marks, DNAse, Motifs changed, Selected eQTL hits |
| rs3774343 | *MLH1* | 1d | 106.39 | Promoter histone marks, Enhancer histone marks, DNAse, Motifs changed, Selected eQTL hits |
| rs969413 | *CEP131* | 1f | 105.32 | Promoter histone marks, Enhancer histone marks, DNAse, Proteins bound, Motifs changed, GRASP QTL hits, Selected eQTL hits |
| rs7567997 | *ADCY3* | 1f | 17.74 | Promoter histone marks, Enhancer histone marks, DNAse, Proteins bound, Motifs changed, GRASP QTL hits Selected eQTL hits |
| rs2286681 | *PMS2* | 1a | 60.12 | Promoter histone marks, Enhancer histone marks, DNAse, Proteins bound, Motifs changed, GRASP QTL hits Selected eQTL hits |
| rs3134866 | *TACC3* | 1f | 204.99 | Promoter histone marks, Enhancer histone marks, DNAse, Proteins bound, Motifs changed, GRASP QTL hits Selected eQTLhits |
| rs28366846 | *USP8* | 1f | 29.11 | Promoter histone marks, Enhancer histone marks, DNAse, Proteins bound, Selected eQTL hits |
| rs4656855 | *CFAP45* | 1f | 34.99 | Promoter histone marks, Enhancer histone marks, DNAse, Proteins bound, Motifs changed, Selected eQTL hits |
| rs11717031 | *TTLL3* | 1f | 106.05 | Enhancer histone marks, DNAse, Proteins bound, Motifs changed, Selected eQTL hits |
| rs2277065 | *KIF3A* | 1a | 201.08 | SiPhy cons, Promoter histone marks, DNAse, Proteins bound, Motifs changed, Selected eQTL hits |
| rs6545790 | *ADCY3* | 1f | 25.95 | Promoter histone marks, Enhancer histone marks, DNAse, Motifs changed, GRASP QTL hits,Selected eQTL hits |
| rs2303426 | *MSH2* | 1f | 202.74 | Promoter histone marks, DNAse, Proteins bound, Motifs changed, Selected eQTL hits |

^a^ Based on Regulome DB Score.

^b^ Based on 3D SNP score

**Supplementary Table 6. Sequences of primers and probes used in this study.**

| **Gene** | **Description** | **Sequences (5'-3')** |
| --- | --- | --- |
| *GAPDH* | Forward | CAATGACCCCTTCATTGACC |
|  | Reverse | TTGATTTTGGAGGGATCTCG |
